# Lipid metabolic reprogramming in colorectal cancer: mechanisms and therapeutic strategies

**DOI:** 10.3389/fimmu.2025.1603032

**Published:** 2025-07-11

**Authors:** Wujianhong Liu, Shengrong Dong, Feiran Hao, Yaohui Gao, Qing Wei

**Affiliations:** Department of Pathology, Shanghai Tenth People’s Hospital Affiliated to Tongji University, Shanghai, China

**Keywords:** lipid metabolism, fatty acids, colorectal cancer, the tumor microenvironment, CRC therapy

## Abstract

Colorectal cancer (CRC) is the third most common cancer worldwide, and its high incidence, mortality, and treatment resistance highlight the urgency of exploring new therapeutic targets. As research into cancer metabolic reprogramming deepens, the central role of lipid metabolism abnormalities in CRC progression has gradually become apparent. In the tumor microenvironment (TME), conditions such as hypoxia, glucose deprivation, and lactic acid accumulation alter the energy demands of tumor cells, driving metabolic reprogramming in lipid uptake, synthesis, and oxidation. This reprogramming helps maintain high energy needs and supports the malignant growth of tumor cells. This lipid metabolic reprogramming provides tumor cells with the necessary energy and enhances their proliferation, invasion, immune evasion, and resistance characteristics. Moreover, the lipid metabolic reprogramming of tumor cells is closely related to various cells within the TME, and these interactions promote, to some extent, the remodeling of the tumor microenvironment, further driving tumor development. Emerging lipid detection technologies position specific lipid molecules as promising biomarkers for auxiliary diagnosis and prognostic evaluation. Concurrently, targeting key lipid metabolic pathways offers innovative strategies to optimize existing therapies and overcome drug resistance. This review summarizes the basic and abnormal mechanisms of lipid metabolism in CRC, lipid metabolic interactions in the tumor microenvironment, the regulatory network between the gut microbiota and lipid metabolism, and the progress in therapeutic strategies targeting lipid metabolism. By exploring the interaction between CRC and lipid metabolism in depth, this review aims to provide new ideas and theoretical support for the treatment, early intervention, and prognosis evaluation of CRC.

## 1 Introduction

CRC is a leading cause of cancer-related mortality globally, with a 5-year survival rate of only 65% (1). In recent years, with changes in lifestyle and population aging, the incidence and mortality rates of CRC have shown an upward trend worldwide, especially in some developing countries where the growth rate is more significant (2, 3). By 2040, there will be 3.2 million new cases and 1.6 million deaths globally (4). Currently, clinical management of CRC relies on a multidisciplinary treatment approach, including surgical resection, adjuvant chemotherapy (such as the FOLFOX regimen), radiotherapy, and targeted therapy (anti-EGFR monoclonal antibodies, anti-VEGF drugs) (5–7). However, these therapeutic strategies still have numerous limitations. First, more than half of early-stage patients experience recurrence or metastasis after surgery (8); second, approximately 23% of CRC patients have metastasis at the time of diagnosis, for whom effective treatments are still lacking. The efficacy of targeted drugs is limited by molecular heterogeneity, such as only about 40% of metastatic CRC patients carrying the wild-type RAS gene benefiting from cetuximab treatment (9); additionally, about 90% of microsatellite stable (MSS) CRC patients do not meet the criteria for immune checkpoint inhibitors (such as PD-1 inhibitors), and more than half of patients with metastatic defective mismatch repair (dMMR) CRC do not respond to immune checkpoint inhibition (ICI) (10). Moreover, during treatment, tumor resistance is a significant concern. Therefore, there is an urgent need to develop new intervention strategies targeting the core biological mechanisms of CRC progression to address current therapeutic challenges.

In recent years, with the continuous advancement of metabolomics and oncology research, metabolic reprogramming has gradually become an important focus in cancer research (11, 12). Tumor cells alter their energy acquisition methods due to their high metabolic demand, preferentially utilizing glycolysis for rapid energy supply through the “Warburg effect,” while also undergoing profound remodeling of lipid and amino acid metabolic pathways (13). This metabolic adaptation not only meets the biosynthetic demands (such as membrane phospholipids and signaling molecules) but also regulates epigenetic modifications, oxidative stress responses, and the immune microenvironment through metabolic products (14). In CRC, metabolic reprogramming exhibits significant stage-dependent dynamics: early-stage tumors primarily activate glycolysis (15), while in advanced stages, tumors display various metabolic abnormalities, with lipid metabolism disorders (such as overexpression of fatty acid synthase, FASN) becoming increasingly prominent (16, 17). Especially when glucose is limited in the microenvironment, tumor cells tend to shift toward lipid metabolism (18). Lipid metabolic reprogramming plays a variety of key roles in the occurrence and development of CRC. On the one hand, excessive fatty acid synthesis provides membrane components and energy reserves for rapid proliferation (19, 20), contributing to tumor cell proliferation; on the other hand, abnormal lipid metabolism can enhance lipid-driven cell signaling (21), contributing to tumor cell proliferation; on the other hand, abnormal lipid metabolism can enhance lipid-driven cell signaling (22–24).

Although intervention strategies targeting lipid metabolism (such as the fatty acid synthase inhibitor TVB-2640) have shown potential in clinical trials, their efficacy is limited by tumor heterogeneity, metabolic pathway redundancy, and the complexity of host-microbiome interactions (25, 26). Therefore, systematically analyzing the dynamic regulatory network of lipid metabolism reprogramming in CRC and elucidating its interactions with the tumor microenvironment (TME) and gut microbiota is an essential step in overcoming the treatment challenges of CRC. In this review, we summarize the key mechanisms of lipid metabolic abnormalities in CRC in recent years, their relationship with the tumor microenvironment and gut microbiota, and discuss therapeutic strategies targeting lipid metabolism, with the aim of providing theoretical support for CRC treatment.

## 2 Lipid metabolism in CRC

**Figure 1:**
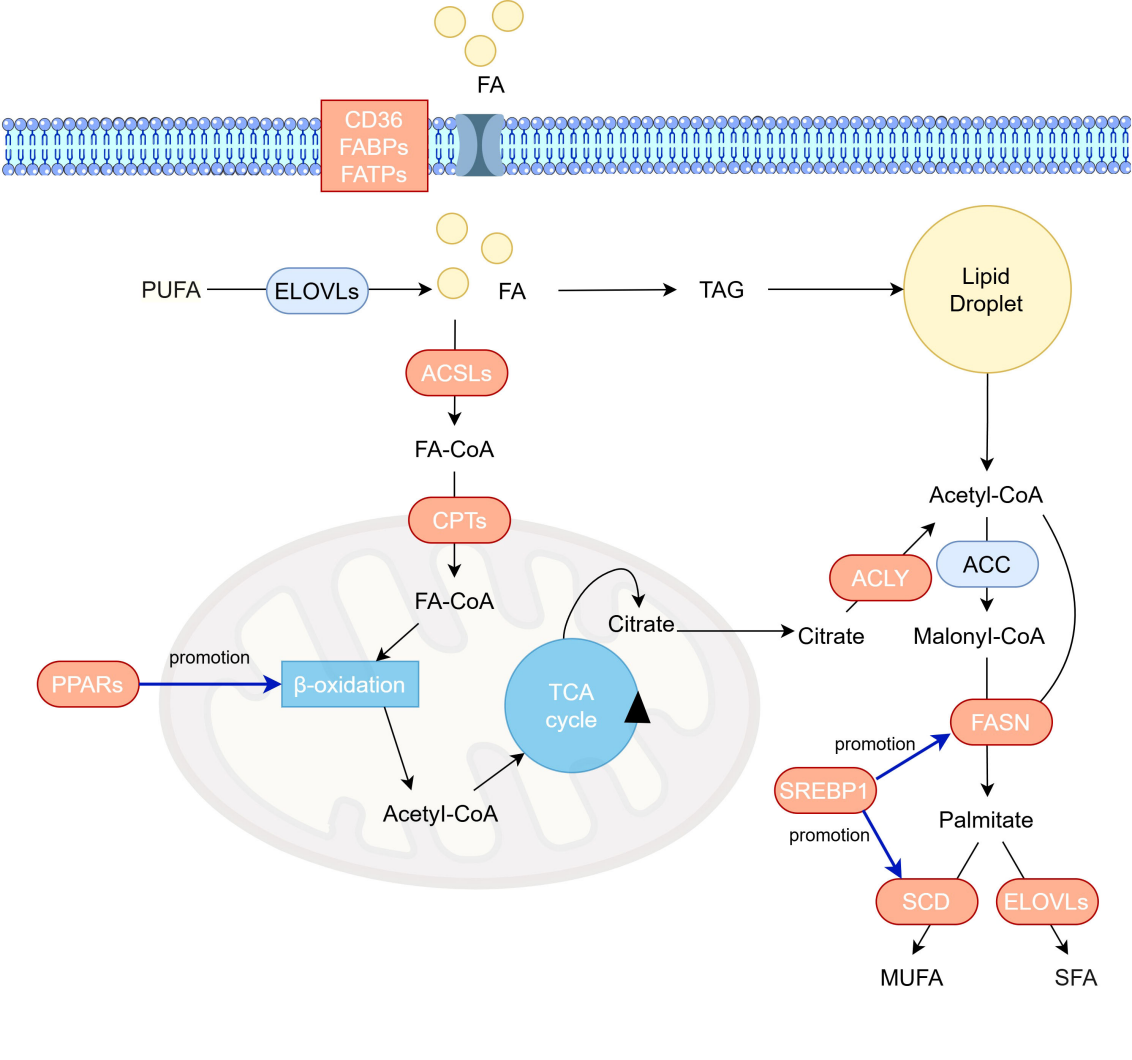
Overview of lipid metabolic reprogramming in colorectal cancer cells. Exogenous fatty acids enter cells via lipid transport proteins (CD36, FABPs, FATPs). Intracellularly, fatty acids are activated to acyl-CoA by ACSL. Long-chain acyl-CoA is then converted to acylcarnitine by carnitine CPT1. After translocation across the inner mitochondrial membrane, carnitine CPT2 regenerates acyl-CoA. Within the mitochondrial matrix, acyl-CoA undergoes β-oxidation to yield acetyl-CoA, which enters the tricarboxylic acid (TCA) cycle for complete oxidation. Citrate from the TCA cycle effluxes to the cytosol, where ACLY catalyzes its conversion to cytosolic acetyl-CoA.ACC subsequently carboxylates acetyl-CoA to malonyl-CoA. Acetyl-CoA and malonyl-CoA are condensed by FASN to form palmitate, which is elongated to 18–24 carbon chains by the ELOVL enzyme family in the endoplasmic reticulum. Activated palmitate can also be desaturated by SCD to generate MUFA and PUFA. Proteins with increased expression in CRC are highlighted in peach. ACC, acetyl-CoA-carboxylase; ACLY, ATP-citrate lyase; ACSL, acyl-CoA synthetase long-chain family; CPT, carnitine palmitoyl transferase; ELOVL, elongation of very-long-chain fatty acids protein; FA, fatty acid; FABP, fatty acid binding protein; FASN, fatty acid synthase; FATP, fatty acid transport protein; SCD, stearoyl-CoA desaturase; SFA, saturated fatty acid; TAG, triacylglycerols; MUFA, monosaturated fatty acid; PUFA, polyunsaturated fatty acid.

Lipid metabolism is a core biological process that maintains cellular homeostasis, encompassing lipid uptake, lipid synthesis, and lipid oxidation (**Figure 1**) (27). At the same mass, lipid metabolism can provide more energy than glycolysis and amino acid metabolism. In CRC cells, lipid metabolism provides energy, builds biological membranes, and serves as secondary messengers to participate in cell activity signaling pathways (28), supporting unique functions such as cell proliferation, growth, invasion, and angiogenesis. **Table 1** summarizing the key factors involved in lipid metabolism in CRC.

**Table 1:** Lipid metabolic pathways and key regulators in CRC.

Pathway	Key factors	Function	Role in CRC	References
Lipid Uptake	CD36	Long-chain fatty acid transporter	Increases proliferation, and invasion; promotes liver/lung metastasis	(37–39)
	FATPs	Fatty acid uptake	Promotes tumor growth and invasion	(41, 43)
	FABPs	Intracellular fatty acid transport	Drives malignant transformation, increases growth, mediates cetuximab resistance	(44–47)
Lipid Synthesis	ACLY	Converts mitochondrial citrate to cytosolic acetyl-CoA	Enhances CTNNB1 activity, cell migration/invasion; induces dormancy via Nanog	(49, 50)
	ACC	Catalyzes acetyl-CoA carboxylation to malonyl-CoA	Supports membrane phospholipid synthesis for rapid proliferation	(51, 52)
	FASN	Synthesizes palmitate from malonyl-CoA	Provides membrane/signaling lipids; activates AMPK/mTOR pathway to increase proliferation; promotes metastasis	(55–57)
	SCD	Converts saturated to monounsaturated fatty acids	Maintains membrane fluidity for metastasis; detoxifies lipids, inhibits ferroptosis	(58–60)
FAO	CPT1A	Transports fatty acids into mitochondria	Provides ATP during starvation; reduces chemo-sensitivity; promotes lung metastasis	(77, 78)
Transcription	SREBP	Activates genes for fatty acid synthesis and cholesterol metabolism	SREBP1: Promotes *de novo* lipogenesis, maintains stemness; SREBP2: Promotes cholesterol synthesis, drives serrated lesion transformation, liver metastasis	(82–84, 86, 87)
	LXR	Regulates cholesterol absorption/efflux	Suppresses c-Myc expression; enhances immune response	(92, 93)
	PPAR	Regulates lipid metabolism/energy homeostasis	PPARα: Enhances FAO and lipid droplets in acid-adapted cells; PPARβ/δ: Upregulates FAO genes, promotes malignant transformation; PPARγ: Suppresses HMGCS2, regulates differentiation, inhibits migration/colony formation	(94–97)

### 2.1 Abnormal lipid uptake in CRC

Lipid uptake is an essential pathway for cells to acquire lipids, mainly involving the uptake of fatty acids (FAs) and cholesterol (29). Rapidly growing tumors require a large amount of lipids. Studies have shown that fatty acid uptake is mainly mediated by fatty acid transport proteins (FATs). Known transport proteins include fatty acid translocase (CD36), fatty acid transport proteins (FATPs), and fatty acid-binding proteins (FABPs) (30).CD36 is a cell surface scavenger receptor that participates in lipid uptake as a lipid transport protein. CD36 expression is upregulated in various tumors such as breast cancer, gastric cancer, and CRC (31–33). Targeted inhibition of CD36 can suppress the growth and metastasis of multiple cancers (34–36), indicating its crucial role in cancer development. In CRC, CD36 expression is significantly higher in tumor tissues compared to adjacent tissues, and CD36 expression is elevated in the liver and non-metastatic lesions (37). Inhibition of CD36 can, to some extent, suppress CRC metastasis (38). Bioinformatics analysis has shown that CD36 regulates cell proliferation and apoptosis in CRC via the PPAR signaling pathway (39). Mechanistic studies have demonstrated that CD36 plays an important role in CRC models. Overexpression of CD36 increases the proliferation, invasiveness, and colony-forming ability of CRC cell lines (HCT116, HT29), upregulates survivin expression, and decreases caspase-3 and PARP cleavage (38). In CD36 knockdown mouse models, the number and volume of lung metastases are significantly reduced after tail vein injection, suggesting that CD36 plays a key role in cell survival and proliferation (37). The FATPs family is another class of key transport proteins, with six homologous proteins (FATP1-6) in the human body. Previous studies have reported that FATPs are upregulated in most cancers, such as breast cancer, melanoma, renal cell carcinoma, and CRC (40–42), and promote tumor growth and invasion by regulating fatty acid uptake (42). In CRC, FATP5 overexpression is particularly significant. Research has shown that it plays a key role in regulating the cell cycle, but its impact on cancer invasiveness remains unclear and requires further investigation (43).FABPs, members of the lipid-binding protein superfamily, are widely involved in intracellular lipid transport and storage. In the APCMin mouse model, knockout of the FABP1 allele reduces the number of adenomas, suggesting the role of this protein in CRC development (44). In *in vitro* models, FABP5 is associated with a more active cell cycle, and knocking down FABP5 leads to cell cycle arrest at the G1 phase and apoptosis (45). FABP4 can enhance cellular lipid metabolism through the AKT pathway and induce intracellular lipid droplet formation (46). Immunohistochemistry shows that FABP4 expression is associated with stronger tumor invasiveness and poorer prognosis (47). Overall, in colorectal cancer, cancer cells can increase fatty acid uptake by upregulating the expression of FATPs, CD36, and FABPs, thereby maintaining the rapid proliferation and growth of tumors (48).

### 2.2 Abnormal lipid synthesis in colon cancer

CRC cells activate abnormal lipid synthesis to meet their rapid proliferation needs. Lipid synthesis is a process centered around acetyl-CoA as a metabolic hub, generating products like fatty acids and cholesterol through various enzymatic reactions. Fatty acid synthesis begins with acetyl-CoA generated in the mitochondria, which is transported to the cytoplasm through the citric acid-pyruvate cycle. In the presence of ATP-citrate lyase (ACLY), acetyl-CoA is converted into cytoplasmic acetyl-CoA. Subsequently, acetyl-CoA carboxylase (ACC) carboxylates acetyl-CoA into malonyl-CoA, which is the rate-limiting step. Fatty acid synthase (FASN) then catalyzes a cyclical process of condensation, reduction (NADPH providing electrons), dehydration, and reduction, ultimately producing palmitic acid (a 16-carbon fatty acid), which is extended to 18–24 carbon chains in the endoplasmic reticulum by the ELOVL enzyme family. Immunohistochemical analysis of CRC patients reveals ACLY overexpression correlating with metastasis. Mechanistic studies demonstrate ACLY stabilizes CTNNB1 (β-catenin) protein, enhancing its transcriptional activity to promote cancer cell migration and invasion (49). Additionally, elevated ACLY expression induces acetyl-CoA accumulation, facilitating P300-mediated H3K27 acetylation to transcriptionally activate Nanog, thereby inducing cellular dormancy in CRC (50). In humans, there are two isoenzymes of ACC, ACC1 and ACC2. ACC1 is significantly overexpressed in CRC tissues, and its activity is aberrantly activated through transcriptional and epigenetic mechanisms. For instance, circular RNA circCAPRIN1 directly binds to the signal transducer and activator of transcription 2 (STAT2), enhancing the transcriptional expression of ACC1, thereby promoting lipid synthesis and driving CRC proliferation and metastasis (51). Mechanistically, the *de novo* fatty acid synthesis mediated by ACC1 is crucial for maintaining tumor cell membrane structure, energy storage, and signal transduction. Knockdown of intestinal epithelial ACC1 significantly reduces the incidence of inflammation-related CRC (52). As one of the key enzymes in lipid synthesis, FASN has been widely studied in CRC (53, 54). Lipidomics research has shown that lipid markers such as mono-unsaturated/poly-unsaturated triglycerides (TG), sphingomyelins, and ceramides are significantly elevated in CRC tissues. FASN gene expression is significantly upregulated and associated with poor prognosis (55). Mechanistic studies have found that FASN can enhance CRC cell proliferation and migration via the AMPK/mTOR pathway (56). Some genes, such as HMGA1, can promote the expression of key lipid synthesizing enzymes like FASN by activating the SREBP1 signaling pathway, significantly increasing the levels of triglycerides and phosphatidylcholine in CRC cells, thus accelerating CRC proliferation and metastasis (57). The rate-limiting enzyme stearoyl-CoA desaturase (SCD) plays an indispensable role in catalyzing the synthesis of monounsaturated fatty acids (mainly oleic acid and palmitoleic acid), both of which are major components of biological membranes. SCD1 is significantly upregulated in cancer and is associated with metastasis and therapeutic resistance. Its core mechanism involves the regulation of fatty acid desaturation and balancing with ferroptosis resistance (58, 59). Inhibiting SCD1 can significantly reduce lipid metabolism and metastasis in CRC cells (60). Due to the demand for long-chain fatty acids in CRC cells, the expression of the fatty acid elongase family (ELOVLs) is usually increased in CRC to meet their demands (61). In conclusion, compared to normal cells, tumor cells exhibit a significantly higher ratio of *de novo* synthesized fatty acids, and this metabolic shift is positively correlated with tumor malignancy (62).

### 2.3 Abnormal lipid storage and oxidation in CRC

Lipid droplets are organelles that store triglycerides and cholesterol, playing a crucial role in maintaining cellular lipid and energy homeostasis (63). Tumor cells mainly store the lipids they intake and synthesize in lipid droplets (64). The increased abundance of lipid droplets is one of the symptoms of cancer aggressiveness (65). Some studies have found that in metastatic cell lines, the total triglyceride and cholesterol content in lipid droplets is elevated, while the content of saturated triglycerides is reduced, suggesting that the degree and ratio of fatty acid storage in lipid droplets are related to tumor invasiveness (66).CRC stem cells are key factors contributing to cancer initiation, drug resistance, and recurrence. The lipid droplet content in CRC stem cells is significantly increased and is directly related to the activity of CD133 and the Wnt signaling pathway, making it one of the markers of stem cells (67). Other studies have shown that *Fusobacterium nucleatum* can induce the acquisition of stemness characteristics in non-colorectal cancer stem cells through lipid droplet-mediated Numb degradation (68). Lipid droplets are also critical in maintaining intracellular stability. Even in the presence of sufficient oxygen, tumor cells are still in an acidic microenvironment due to the excessive production of lactic acid (69). Chronic acidic microenvironments lead to mitochondrial remodeling in tumor cells to maintain energy production, and this remodeling depends on the buffering system of lipid droplets (70). Fatty acids generated by autophagy are directed into lipid droplets to prevent lipid toxicity to mitochondria. Inhibition of lipid droplet formation disrupts mitochondrial function and impairs the transport of fatty acids to mitochondria (71, 72). Furthermore, there is a close interaction between lipid droplet formation and fatty acid oxidation (FAO), which together maintain the metabolic balance of cancer cells (72).

Fatty acids exhibit high energy density, yielding approximately 9 kcal per gram upon oxidation—more than double the energy derived from glucose. Under nutrient-replete conditions, certain malignancies preferentially utilize fatty acid oxidation for energy production (73, 74).FAO generally involves four stages: activation, transfer, β-oxidation, and complete oxidation. In the cytoplasm, fatty acids are catalyzed by acyl-CoA synthetase to form activated acyl-CoA, consuming ATP in the process. Then, long-chain acyl-CoA enters the mitochondria through the carnitine shuttle system: carnitine palmitoyltransferase I (CPT1) converts it into acylcarnitine, which is transported across the mitochondrial inner membrane, and carnitine palmitoyltransferase II (CPT2) regenerates it into acyl-CoA. This process is the rate-limiting step. In the mitochondrial matrix, acyl-CoA undergoes β-oxidation to produce acetyl-CoA, which then enters the citric acid cycle and is fully oxidized to generate large amounts of ATP (75). A study integrating CRC bulk and single-cell transcriptomic data and using GFAO_Score to represent FAO levels found that the high GFAO_Score group had higher staging and decreased sensitivity to chemotherapy drugs (76). The rate-limiting enzyme of FAO, CPT1, has three isoforms (CPT1A-C). Studies targeting CPT1A have shown that, compared to the primary site, the expression level of CPT1A in metastatic sites is significantly increased, and inhibiting CPT1A expression can reduce the lung metastatic rate of CRC. Another study targeting CPT1C found that inhibiting CPT1C can suppress CRC cell FAO, proliferation, and migration (77). Compared to normal tissues, CRC significantly increases the likelihood of peroxidation due to oxidative stress (78). Some lipid peroxidation products, such as epoxy-ketone eicosatrienoic acid (EKODE), accumulate in CRC cells and induce an inflammatory response in colonic epithelial cells by activating the JNK pathway, promoting tumor progression in the AOM/DSS-induced CRC mouse model (79).

### 2.4 Transcriptional regulation of lipid metabolism in CRC

The transcriptional regulation of lipid metabolism in CRC is a complex network of multi-factor collaboration, with several key transcription factors at its core. Sterol regulatory element-binding protein (SREBP) is a central transcription factor in regulating lipid metabolism. It promotes *de novo* lipid synthesis in CRC cells by activating the expression of genes involved in fatty acid synthesis and cholesterol metabolism, playing a crucial role in the occurrence, development, and metastasis of CRC (80). The SREBP family comprises two homologous genes, SREBP1 and SREBP2. SREBP1 primarily regulates fatty acid and triglyceride synthesis, whereas cholesterol biosynthesis is predominantly governed by SREBP2 (81). Knockdown of SREBP in CRC cell lines significantly reduces intracellular fatty acids, cholesterol, and triglycerides, inhibiting tumor cell proliferation. Further bioenergetics analysis shows that SREBP knockdown inhibits mitochondrial respiration, glycolysis, and FAO, leading to an overall metabolic shift in the cells. This metabolic remodeling results in a significant reduction in cancer cell proliferation and weakens its ability to form tumor spheroids, suggesting that SREBP-dependent lipid synthesis is critical for maintaining the stemness characteristics of CRC cells (82). Analysis of GEO tissue microarrays revealed that the expression of long non-coding RNA (lncRNA) ZFAS1 is upregulated in CRC. ZFAS1 stabilizes SREBP1 mRNA by binding to poly(A)-binding protein 2, allowing its accumulation and reprogramming lipid metabolism (83). Studies on PIK3CA mutations and tumor lipid metabolism reveal that the PIK3CA-E545K mutation promotes nuclear accumulation of SREBP1, enhancing transcription of apolipoprotein A5 (APOA5) and thereby mediating platinum-based drug resistance in CRC (84). Precursors of SREBPs (pre-SREBPs) bind to their partner SREBP cleavage-activating protein (SCAP) and reside in the endoplasmic reticulum. Upon cellular cholesterol depletion, the SREBP/SCAP complex translocates to the Golgi apparatus, where SREBP undergoes proteolytic cleavage. The N-terminal SREBP fragment then enters the nucleus and binds sterol regulatory elements (SREs) in promoter regions of target genes, upregulating key enzymes for cholesterol synthesis (e.g., HMG-CoA reductase, squalene monooxygenase) (85). This transcriptional reprogramming drives excessive *de novo* cholesterol synthesis and uptake, providing essential membrane components, lipid raft structures, and signaling molecule precursors for rapid cancer cell proliferation. Research on CRC metastasis demonstrates significantly elevated SREBP2 expression in liver metastases compared to primary tumors—a phenomenon not observed in brain or lymph node metastases—indicating pathway-specific activation of cholesterol synthesis in hepatic metastasis. SREBP2 knockdown markedly reduces both the number and volume of liver metastases in nude mice (86). In aPKC-deficient intestinal tumors, enhanced SREBP2 activation upregulates cholesterol biosynthesis, promoting cellular metaplasia and the formation of aggressive cellular subsets within serrated tumor lesions (87).

Liver X receptors (LXR) are another type of transcription factor that regulates lipid metabolism. There are two subtypes in humans, LXRα and LXRβ. These are encoded by different genes but have similar functions (88). In the intestinal epithelium, LXR acts as a cholesterol sensor, regulating cholesterol absorption and excretion. It negatively regulates the expression of cholesterol uptake protein Niemann-Pick C1-like 1 (NPC1L1), thereby reducing intestinal cholesterol absorption (89). At the same time, LXR induces the expression of ATP-binding cassette transporters ABCG5 and ABCG8 to promote fecal cholesterol excretion (90). This action helps maintain systemic cholesterol homeostasis and is associated with a reduced risk of CRC development. Clinical CRC cohorts further verified this role: LXR expression is decreased in CRC patients, and the tissue cholesterol content is elevated (91). LXR activation can inhibit the activity of β-catenin, a key transcription factor in the Wnt/β-catenin signaling pathway, which is frequently dysregulated in CRC. By inhibiting β-catenin, LXR reduces the expression of oncogenes such as c-Myc, thereby suppressing CRC proliferation (92). Additionally, studies have shown that in mice fed with the LXR receptor agonist GW3965, the number of tertiary lymphoid structures in tumors increases, adaptive immune responses are enhanced, and tumor formation is reduced (93).

Peroxisome proliferator-activated receptors (PPARs) play pivotal regulatory roles in the lipid metabolism of CRC. Their three subtypes (PPARα, PPARβ/δ, PPARγ) influence tumorigenesis, progression, and metabolic reprogramming through distinct mechanisms. PPARα modulates fatty acid mobilization and enhances FAO. Elevated PPARα expression in CRC exhibits dual tumor-suppressive and oncogenic roles. In PPARα-deficient murine colon cancer models, increased levels of DNMT1 and PRMT6 promote colon carcinogenesis by mediating methylation of p21 and p27, respectively; the PPAR agonist fenofibrate suppresses AOM/DSS-induced colorectal carcinogenesis (94). Conversely, within acidic tumor microenvironments, upregulated PPARα fuels cancer cell proliferation and invasion by enhancing FAO and lipid droplet (LD) accumulation, thereby supporting the energy demands of acid-adapted CRC cells (95). Under high-fat diet conditions, activated PPARβ/δ induces downstream FAO gene expression, potentiates intestinal stem cell function, and elevates tumorigenic risk (96). As a regulator of adipogenesis, PPARγ activation negatively modulates mitochondrial HMGCS2 to govern intestinal cell differentiation (97). PPARγ also serves as a receptor for linoleic acid, suppressing CRC cell migration and colony formation upon linoleic acid treatment (98).

## 3 Lipid metabolism interactions in the tumor microenvironment

**Figure 2:**
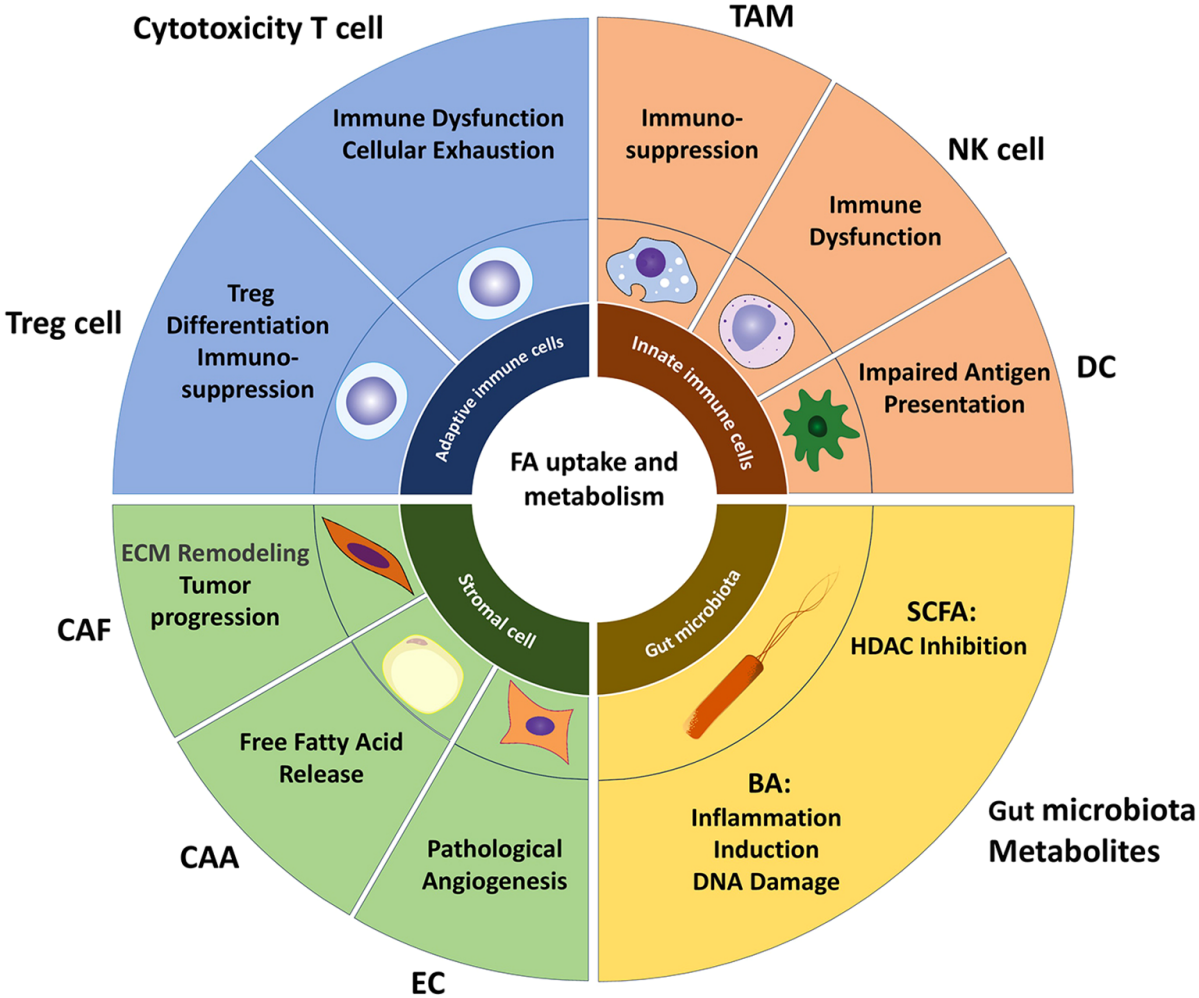
Lipid metabolism landscape in TME. Lipid metabolism within the TME establishes a multi-layered pro-tumorigenic network by regulating immune cell phenotypes/functions and promoting angiogenesis. Tumor-infiltrating cytotoxic T cells commonly exhibit metabolic dysfunction, where excessive lipid accumulation impairs T cell function and promotes exhaustion. Fatty acids facilitate Treg differentiation by suppressing lineage-defining transcription factors while upregulating FoxP3 expression. The majority of TAM adopt an immunosuppressive M2 phenotype, which internalizes free fatty acids (FFAs) released by tumor cells via extracellular vesicles and CD36, reinforcing their immunosuppressive activity. Intracellular lipid accumulation compromises NK cell cytotoxicity against tumors and diminishes antigen-presenting capacity in DCs. Among stromal cells, enhanced lipid metabolism supplies lipids to tumors, remodels the extracellular matrix, and induces angiogenesis, collectively driving tumor proliferation and metastasis. Microbial metabolites SCFAs and BAs—play pivotal roles in the TME: SCFAs accumulate in CRC cells, acting as HDAC inhibitors to suppress proliferation, while BAs promote CRC development by inducing inflammation and DNA damage. TAM, tumor-associated macrophage; DC, dendritic cell; CAF, cancer-associated fibroblast; CAA, cancer-associated adipocyte; EC, endothelial cell; SCFA, short-chain fatty acid; BA, Bile acid.

The TME is a complex system composed of tumor cells, immune cells, stromal cells, and non-cellular components (**Figure 2**). Its interaction with metabolism can significantly impact tumor progression and therapeutic resistance (99, 100). Recent studies have found that lipid metabolism in the TME not only provides energy and biosynthetic precursors for tumor cells but also forms a multi-layered pro-cancer network by regulating immune cell phenotype and function, promoting angiogenesis, and maintaining cancer stem cell characteristics (68, 101, 102).

### 3.1 Immune cells and lipids

Hypoxia, acidic environments, and lipid accumulation in the tumor microenvironment can suppress the function of immune cells within it (102). Immune cells in the TME also undergo metabolic changes to maintain certain functions (103). Immune cells in the TME can be categorized into adaptive immune cells and innate immune cells. T cells are the primary adaptive immune cells in the tumor microenvironment. Within the TME, T cells are mainly divided into two categories: one consists of effector T cells and helper T cells, which inhibit tumor growth; the other consists of regulatory T cells (Tregs), which suppress immune responses (104). Studies have shown that tumor-infiltrating T cells often exhibit metabolic disorders, characterized by enhanced metabolic switching from glycolysis to lipid oxidation (105, 106). However, excessive lipid metabolism can affect T cell function and lead to T cell exhaustion (107); linoleic acid increases CPT1A in CD4+ T cells, leading to oxidative stress and inducing apoptosis (108); linoleic acid increases CPT1A in CD4+ T cells, leading to oxidative stress and inducing apoptosis (109); excessive lipids in TAM can also induce an increase in CD36 expression in CD8+T cells, leading to lipid peroxidation and functional impairment of the cells (110); at the same time, persistent antigen stimulation in the TME can induce the gradual loss of PGC1α in CD8+ T cells via the Akt signaling pathway, affecting mitochondrial replication, gradually reducing the cells’ ability to utilize lipids, and triggering CD8+ T cell exhaustion (111). Treg cells mainly rely on FAO for energy (112).In hypoxic conditions with lactate accumulation in the TME, Treg cells can upregulate CD36 expression and adjust mitochondria through the PPAR-β signaling pathway to maintain their function. Knocking out Treg cell CD3 can reduce the expression of various immune regulatory receptors (113). Using sulfonated N-succinimide oleic acid (SSO) and etomoxir to inhibit fatty acid transport and oxidation can decrease the number of Treg cells and the expression of cell markers (114).

In the tumor microenvironment, innate immune cells mainly include macrophages, NK cells, and dendritic cells. Macrophages are considered one of the main cell types leading to lipid metabolic disorders in the TME. Compared with normal samples, the expression of ALOX5 is increased and the pro-inflammatory lipid product 5-HETE is significantly up-regulated in CRC. Analysis of large Affymetrix microarray datasets shows that genes related to arachidonic acid lipid metabolism and pro-inflammatory mediators are co-expressed with relevant macrophage markers, indicating the important role of macrophages in driving pro-inflammatory responses and lipid disorders in the TME (115). Tumor-associated macrophages (TAMs) are primarily divided into the pro-inflammatory M1 type and the immunosuppressive M2 type (116). M1 macrophages rely on glycolysis, while M2 macrophages obtain energy through FAO. M1 macrophages kill tumor cells through antibody-mediated cytotoxicity, but during this process, the glucose uptake demand of the cells significantly increases. The lack of glucose and the accumulation of lactate in the TME induce the transformation of M1 macrophages into M2 type (117). Therefore, most of the macrophages in the tumor microenvironment are immunosuppressive M2 type (118). M2 macrophages have enhanced fatty acid uptake and synthesis abilities and rely on FAO for energy supply in the TME (119). Free fatty acids released by tumor cells are extensively taken up by M2 macrophages through extracellular vesicles and CD36, promoting their immunosuppressive phenotype (120). This suppressive ability is mainly reflected in the inhibition of CD8+ T cell function, and inhibition of CD36 can reduce M2 polarization and enhance CD8+ T cell function (120). Additionally, oleic acid in the environment can lead to lipid droplet accumulation in infiltrating macrophages in colon cancer and promote M2 polarization via activation of the mTOR signaling pathway. Injection of lipid droplet synthesis inhibitors can suppress tumor growth *in vivo* models (121). Single-cell transcriptomics reveals a terminally differentiated C5 macrophage subset characterized by robust lipid metabolic reprogramming and potent immunosuppressive function (122). A study on obesity found that the number and function of NK cells in obese patients are impaired, and adiponectin combined treatment can restore part of the NK cell function, suggesting that lipid metabolism can affect NK cells (123). Research has shown that in a high cholesterol microenvironment, CRC cells upregulate TGF-β1 secretion through ATP6V0A1-dependent cholesterol uptake, indirectly inhibiting the immune surveillance function of NK cells (124). Another study found that lipid accumulation after colorectal cancer surgery leads to increased CD36 and lipid content in NK cells, impairing their cytotoxic function against tumors (125).In the colorectal cancer tumor microenvironment, lipid metabolism reprogramming of dendritic cells (DCs) significantly affects their immune function and anti-tumor response. Single-cell transcriptomic analyses reveal significant enrichment of lipid metabolism, fatty acid metabolism, and PPARA signaling pathways in DCs. Concurrently, the transcription factor RUNX2 is markedly upregulated in tumor-infiltrating DCs, where it orchestrates downstream PPAR signaling via activation of the Wnt/β-catenin axis, thereby remodeling cellular lipid metabolism (126). The research found that lipid droplets containing electrophilic oxidized truncated (ox-tr) lipids in tumor DCs were much larger than in the control group. These lipid droplets covalently bind to heat shock protein 70, hindering MHC translocation, and resulting in a decrease in the antigen presentation ability of DCs (127). Using ACC inhibitors to reduce intracellular lipids in DCs can partially restore their function, indicating that lipid metabolism reprogramming in the TME is an important mechanism affecting DC function (128).

### 3.2 Adipocytes

The intestine is an organ rich in fat, and CRC establishes intimate connections with adipocytes when it invades adipose tissue (129). CRC cells induce adjacent adipocytes to dedifferentiate into cancer-associated adipocytes (CAAs) through direct contact or paracrine signaling. These CAAs are characterized by a reduction in lipid droplets, enhanced lipolysis, and increased secretion of pro-inflammatory factors (130, 131). Co-culture experiments show that CRC cells inhibit the expression of genes related to adipogenesis (such as FADS1, and SC4MOL) and activate lipolytic pathways, leading to the release of free fatty acids and cholesterol from adipocytes (130). These lipids are taken up by tumor cells via transport proteins such as CD36, providing energy and membrane synthesis materials for tumor cells (132). These fatty acids can also activate the AMPK signaling pathway, inducing autophagy and promoting the transformation of colon cancer into a mode of energy acquisition primarily based on FAO (133). Adipocytes also enhance the expression of FABP4 in surrounding tissues and mediate resistance to cetuximab (134). CAAs promote CRC cell proliferation and metastasis by secreting pro-inflammatory factors such as TNF-α and LCN2, activating the YAP/TAZ signaling pathway (131). Additionally, exosomes secreted by colon cancer cells, containing miR-146b-5p, induce the browning and lipid mobilization of white adipocytes, which is one of the causes of cachexia (135). Similarly, in colorectal cancer chemotherapy models, first-line chemotherapy drugs reduce the expression of proteins involved in ATP generation, β-oxidation, and lipid synthesis in adipocytes, leading to adipose depletion (136). This depletion of fat is associated with shortened survival and quality of life, warranting more research and attention (137).

### 3.3 Cancer-associated fibroblasts

Cancer-associated Fibroblasts (CAFs) are key components of the TME and interact with colorectal cancer through the secretion of cytokines, remodeling the extracellular matrix (ECM), and regulating metabolic pathways, including lipid metabolism (138, 139). CAFs upregulate the expression of genes related to fatty acid synthesis and secrete large amounts of fatty acids and phospholipids. After being taken up by CRC cells, these lipids enhance tumor cell migration ability (140). CRC cells also transfer the HSPC111 protein via exosomes to hepatic stellate cells, converting them into CAFs and preparing for liver metastasis. Furthermore, HSPC111 can phosphorylate ACLY, increasing the acetyl-CoA level in CAFs and promoting the expression and secretion of the chemokine CXCL5 through H3K27 acetylation in an epigenetic manner. The secreted CXCL5 activates the epithelial-mesenchymal transition (EMT) of CRC cells through the CXCR2 receptor, forming a positive feedback loop that further stimulates the release of HSPC111 in tumor exosomes (141). Spatial transcriptomics analysis has found that lipid metabolism-related pathways are significantly upregulated in areas enriched with inflammatory cancer-associated fibroblasts (iCAFs). In iCAF-enriched regions of patients receiving neoadjuvant chemotherapy, despite an overall decrease in metabolic activity, fatty acid metabolic activity remains high, suggesting that iCAFs may promote chemotherapy resistance by maintaining lipid metabolic activity (142).

### 3.4 Endothelial cells

In CRC, endothelial cells (ECs) are the central executors of tumor angiogenesis. In the tumor microenvironment, due to a lack of glucose, proliferating endothelial cells undergo metabolic reprogramming and switch to fatty acid oxidation for energy production (143). In this state, endothelial cells exhibit increased lipid uptake and enhanced expression of FABP4, and inhibition of FABP4 can reduce tumor angiogenesis (144). Studies have shown that vascular endothelial growth factor B regulates endothelial cell fatty acid uptake through the vascular fatty acid transporters, although the exact mechanism remains unclear (145). Co-culture experiments have revealed that FASN affects endothelial cell activity by regulating the secretion profile of angiogenic factors. Knocking down FASN significantly reduces microvessel density in colon cancer cell lines and induces the “normalization” of vascular structures. Overexpression of FASN enhances MMP-9 activity and VEGF secretion, promoting endothelial cell activation (146). Other studies have shown that inhibiting CRC lipid metabolism through PI3K inhibitors reduces angiogenesis, suggesting the important role of lipid metabolism in tumor vascular formation (147). Single-cell transcriptomics of obese CRC samples reveal enrichment of an ESM1^+^ EC subpopulation in tumors, correlating with poorer prognosis. This subpopulation exhibits upregulated pathways regulating epithelial cell migration and adhesion, indicating a pro-tumorigenic role (148).

### 3.5 Gut microbiota and the regulatory network of lipid metabolism

The occurrence of CRC is closely related to various factors, among which the interaction between gut microbiota and lipid metabolism is considered to play a significant role in the development of CRC (149, 150). The gut microbiota interacts with host lipid metabolism through metabolic products such as short-chain fatty acids (SCFAs) and secondary bile acids, forming a complex regulatory axis. SCFAs, such as acetate, propionate, and butyrate, are the primary metabolites produced by gut microbes through the fermentation of dietary fibers (151). The benefits of butyrate are well known, as it can regulate gut immune responses by activating G-protein-coupled receptors (such as GPR43 and GPR109A), reduce chronic inflammation, and thus decrease the risk of carcinogenesis (152). In the TME, butyrate can also enhance CD8+ T cell responses through IL-2-related signaling pathways, promoting the antitumor effects of PD-1 inhibitors (153). Butyrate also induces metabolic adaptations in activated CD8^+^ T cells, redirecting TCA cycle substrates toward fatty acid uptake and FAO (154). Butyrate metabolism diverges between normal intestinal epithelium and cancer cells: Normal epithelia utilize butyrate via β-oxidation, whereas CRC cells exhibit diminished catabolic capacity due to the Warburg effect, leading to intracellular accumulation (155). Once accumulated, butyrate acts as a histone deacetylase (HDAC) inhibitor, leading to CRC cell apoptosis. Metabolically, butyrate binds to pyruvate kinase M2 (PKM2), promoting its dephosphorylation and tetramerization. This activates PKM2, suppresses the Warburg effect, reduces glycolytic energy production, and inhibits CRC growth (156). Chronic butyrate exposure diminishes glycolytic capacity in CRC cells regardless of glucose availability. Butyrate increases mitochondrial pyruvate oxidation flux, thereby enhancing *de novo* lipid synthesis and lipid accumulation (157). Additionally, studies report upregulated SLC27A1 (FATP) expression in butyrate-treated CRC cells, augmenting fatty acid transport (158). Short-chain fatty acids (SCFAs) and their derived acyl-CoAs can also be oxidized. Under hypoxia, tumor cells convert acetate to acetyl-CoA via ACSS2, activating lipogenic genes (e.g., FASN) to fuel *de novo* lipogenesis and support growth (159). Conversely, CD8^+^ T cells utilize acetate in an ACSS2-dependent manner to rescue effector functions impaired by glucose deprivation (160), highlighting acetate’s paradoxical roles in tumor progression. Acetate is converted to butyrate in the gut by bacteria such as Roseburia spp. Via butyryl-CoA:acetyl-CoA transferase (161). Propionate, as an HDAC inhibitor, can synergize with butyrate to inhibit tumor cell proliferation, although its potency is relatively weaker (162). Furthermore, recent studies have shown that SCFAs, in addition to their HDAC inhibition effects, can induce propionylation modifications at histone H3K18 and H4K12 sites, inhibit the Wnt/β-catenin signaling pathway, upregulate key CRC oncogenes (such as MYC, FOS, and JUN), and downregulate genes related to cell proliferation (ANP32B) and the cell cycle (MKI67), leading to CRC cell death (163).

Bile acids (BA) and their derivatives are another type of metabolite produced by the gut microbiota, which plays a dual role in the development of CRC (164). Primary BAs, such as cholic acid (CA) and chenodeoxycholic acid (CDCA), are produced in the liver and can be converted into secondary BAs by the gut microbiota. Some secondary BAs, such as deoxycholic acid (DCA) and lithocholic acid (LCA), have been shown to promote CRC by inducing inflammation, DNA damage, and cell proliferation (165). However, other BAs, such as ursodeoxycholic acid (UDCA), may have protective effects by reducing inflammation and inhibiting tumor growth via the TGR5-YAP axis (166).

The gut microbiota not only exerts significant effects on the intestinal microenvironment through its metabolites but also influences the progression of CRC by regulating lipid metabolism pathways. In the Apcmin/+ mouse model, a high-fat diet affects phospholipid metabolism via the gut microbiota, leading to intestinal mucosal damage and CRC development (167).In obese CRC patients, the dysregulation of fatty acid and phospholipid metabolism is closely associated with the enrichment of pathogenic bacteria and the reduction of beneficial bacteria (168). Additionally, the intrinsic lipid metabolism reprogramming in colorectal cancer also affects the gut microbiota. The rate-limiting enzyme in cholesterol biosynthesis, squalene epoxidase (SQLE), is significantly upregulated in CRC (169). Overexpression of SQLE indirectly accelerates tumor progression through gut microbiota dysbiosis: the gut microbiota of SQLE transgenic mice shows enrichment of pathogenic bacteria (such as C. muridarum), reduced abundance of anti-inflammatory and protective bacteria (such as S. violaceusniger), elevated levels of secondary bile acids (such as DCA), reduced tight junction proteins, and impaired intestinal barrier function, which aggravates CRC progression (170).

## 4 Lipid metabolism-related biomarkers and analytical techniques in CRC

Lipids serve as fundamental components of cellular membranes and participate in diverse metabolic processes, playing critical physiological roles (171). During the occurrence of CRC, lipid metabolism shows significant disorders, manifested as abnormal expression and dysfunction of multiple lipid molecules and related proteins. These changes directly drive the malignant biological behavior of tumors and become potential diagnostic and prognostic biomarkers. With the rapid development of lipidomics technologies, especially the application of mass spectrometry-based high-throughput analysis platforms combined with multivariate statistical methods, complex lipid quantitative analysis in cancer has become possible, and more and more new lipid biomarkers have been discovered thereby.

### 4.1 Lipid metabolism-related biomarkers in CRC

Characteristic lipidomic remodeling occurs during CRC development. Profiling these distinct alterations provides valuable insights for CRC diagnosis and prognosis. CRC patients exhibit significant imbalances in fatty acid composition within serum and tumor tissues, including elevated long-chain PUFAs (e.g., arachidonic acid [AA], eicosapentaenoic acid [EPA], docosahexaenoic acid [DHA]) and reduced levels of PUFAs such as linoleic acid (LA) and α-linolenic acid (ALA) (172). Comparative analyses reveal upregulated ω-6 PUFAs in carcinomas versus adenomas, while ω-3 PUFAs are downregulated in CRC (173). Additionally, decreased monounsaturated fatty acid (MUFA) content alongside elevated saturated fatty acids (SFAs) and n-3/n-6 PUFAs has been observed in CRC tissues (174). Assessing these FA profiles may aid in early CRC detection. β-Hydroxybutyrate (BHB), a ketone body derived from FAO, demonstrates progressively increased serum levels across CRC stages, potentially serving as a biomarker for tumor metabolic reprogramming (175). Notably, when administered as part of a ketogenic diet, BHB suppresses CRC growth (176), warranting further investigation into its context-dependent functions. Associations between triglycerides (TAGs) and CRC risk remain inconsistent: A Chinese cohort study identified elevated TAG levels as positively correlated with colon cancer progression (177), whereas analysis of the UK Biobank found no statistically significant TAG-CRC association after adjusting for BMI and other factors (178). The dietary variations influence TAG measurements (55). Phosphatidylethanolamine (PE) enrichment in plasma exosomes distinguishes non-metastatic CRC patients from healthy controls, with lower PE levels observed in metastatic versus non-metastatic cases (179). Such lipidomic shifts reflect functional membrane alterations across CRC stages.

Given CRC lipid complexity, multi-lipid biomarker panels outperform single molecules for staging. A diagnostic panel comprising O-(4,8-dimethylnonanoyl)carnitine, LPC 19:0, TAG 58:1, and PC 38:7 achieves an AUC of 0.805 (95% CI: 0.684–0.922) for discriminating adenomas from CRC (180). Krishnan et al. constructed machine learning models utilizing ceramide (CE)(22:6), CE(18:3), TG(56:9), and FA combinations to identify liver metastasis in CRC (17). Liu et al. developed an integrated model (ApoA1, ApoA2, lithocholic acid [LCA], CEA) with an AUC of 0.995 (95% CI: 0.969–0.999) for CRC diagnosis (181).

### 4.2 Lipid analysis techniques in CRC

With the development of high-throughput technologies, lipidomics has gradually matured in CRC research and biomarker discovery. The main analytical tools currently include mass spectrometry (MS) and nuclear magnetic resonance (NMR). MS plays a core role in the analysis of lipid metabolic changes in CRC (182). MS-based lipid analysis encompasses non-imaging and imaging strategies. In non-imaging methods, gas chromatography-mass spectrometry (GC-MS) is suitable for analyzing volatile small-molecule lipids (molecular weight < 1000 Da). It has high sensitivity but limited coverage (183). Liquid chromatography-mass spectrometry/capillary electrophoresis-mass spectrometry (LC-MS/CE-MS) is widely used for complex lipids (such as phospholipids and glycerolipids), serving as the core tool of lipidomics. It can qualitatively and quantitatively analyze hundreds of lipids, especially suitable for global analysis of biological fluids (serum, urine) and tissue extracts (115, 184).

Imaging methods focus on spatial resolution analysis, providing direct information on the spatial distribution of target molecules, but compared with high-performance liquid chromatography-mass spectrometry (HPLC-MS), the analytical depth is reduced (185). Matrix-assisted laser desorption/ionization mass spectrometry imaging (MALDI-MSI) achieves lipid ionization through matrix co-crystallization, with a spatial resolution of 5–200 μm. It can be coupled with high-resolution mass spectrometry such as Fourier transform ion cyclotron resonance (FTICR) to distinguish lipid isomers, but requires optimization of matrices (such as 2,5-dihydroxybenzoic acid, α-cyano-4-hydroxycinnamic acid) and deposition techniques to suppress ion interference (186, 187); Desorption electrospray ionization mass spectrometry imaging (DESI-MSI) directly analyzes tissue sections in an open environment, suitable for rapid clinical detection (such as intraoperative diagnosis), with a resolution of approximately 50–200 μm (nano-DESI can reach < 10 μm) (188, 189); Secondary ion mass spectrometry (SIMS) must be performed under vacuum, with a resolution up to the nanometer scale (0.05–100 μm), suitable for single-cell lipid distribution research, but it is destructive to samples and can cause lipid fragmentation (190). Multimodal integrations (e.g., LIMS with infrared spectroscopy/spatial transcriptomics) and emerging single-cell LIMS (e.g., SpaceM) provide multidimensional insights into lipid metabolism across molecular, cellular, and tissue levels, advancing mechanistic and clinical applications (188). NMR spectroscopy features non-destructive analysis and simple sample preparation. Compared to MS, NMR has a weaker resolution for similar lipids and is currently mainly used for non-targeted analysis (191). For example, Mika et al. employed ¹H-NMR for global lipid profiling followed by GC-MS for major component analysis in CRC (174).

## 5 Targeting lipid metabolism in CRC treatment strategies

As summarized above, lipid metabolism plays a crucial role in the occurrence and development of CRC. These findings have led us to consider lipid metabolism as a potential target for intervention in order to impede the progression and drug resistance of CRC. Currently, therapeutic strategies targeting metabolic vulnerabilities have shown promising translational potential.

### 5.1 Targeting lipid metabolism in treatment

Inhibiting the *de novo* lipogenesis (DNL) pathway has become an important strategy for cancer treatment (192).FASN, the rate-limiting enzyme in DNL, is closely associated with tumor cell proliferation, metastasis, and chemotherapy resistance, making it a key target for inhibiting lipid synthesis (54, 56). Cerulenin, the first-generation FASN inhibitor, can inhibit tumor cell energy metabolism and the mTOR signaling pathway, thereby inhibiting CRC cell proliferation (193). In mice, Cerulenin inhibits CRC liver metastasis and reduces the size of liver metastatic lesions (194). Epigallocatechin gallate(EGCG), a green tea extract, exerts strong FASN inhibition, which reduces the levels of free fatty acids in tumors and decreases tumor volume in xenografted mice, without affecting the mice’s weight (195). Randomized clinical trials have found that this extract can significantly reduce the incidence of colon adenomas (RR, 0.56; 95% CI, 0.34-0.92) (196). Luteolin, a flavonoid compound, exerts anticancer effects by regulating the Wnt/β-catenin pathway and the miR-384/PTN axis (197, 198). TVB inhibitors, a new generation of potent FASN inhibitors, including TVB-2640 and TVB-3664, have shown good results in Phase I clinical trials (25, 199). In xenograft models derived from CRC patients, TVB-3664 reduced tumor lipid storage and inhibited tumor growth in 30% of the samples. Additionally, Orlistat, as a sulfatase domain inhibitor, has shown antitumor activity in breast cancer and CRC models, but its use in clinical settings is limited by its gastrointestinal carcinogenic effects (200). It is worth noting that CRC cells can counteract the effects of FASN inhibitors by upregulating CD36. In this case, the combination of CD36 inhibitors can enhance the efficacy of FASN inhibitors (38).

Apart from FASN, other lipid synthesis proteins can also be targeted. ACLY can generate cytosolic acetyl-CoA and bridge the mitochondria and non-mitochondrial processes. The ACLY inhibitor SB204990 inhibits acetyl-CoA generation, suppressing tumor cell proliferation and inducing differentiation (201). ACC allosteric inhibitors, such as TOFA, can inhibit fatty acid synthesis and induce CRC cell apoptosis in a dose-dependent manner (202). SCD1, the rate-limiting enzyme in unsaturated fatty acid synthesis, is highly expressed in CRC (58). Its inhibitor, Betulinic Acid, can impair CRC stem cell clonogenic ability and induce cell death (203). Notably, targeting the DNL pathway requires balancing therapeutic efficacy and metabolic toxicity. For example, systemic inhibition of ACC may induce thrombocytopenia, while local delivery technologies (such as nanoparticle encapsulation) are being explored to enhance selectivity (192).

Targeting lipid oxidation is also an important strategy for CRC therapy. CPT1 is the rate-limiting enzyme in mitochondrial fatty acid transport (75). Its inhibitor Etomoxir can significantly inhibit fatty acid oxidation, inducing an energy crisis in CRC cells (204). Another inhibitor, DHP-B, extracted from Piperaceae plants, can disrupt the interaction between mitochondrial CPT1A and VDAC1, leading to increased mitochondrial permeability and reduced energy synthesis, thereby inhibiting CRC cell growth and inducing apoptosis (77).

**Table 2** summarizes ongoing or completed clinical studies related to lipid metabolism.

**Table 2:** The clinical research on targeting lipid metabolism in CRC.

NCT number	Main target	Compound	Stage of clinical trial	Comments
NCT01606124	FASN	EGCG	phase II	No significant reduction in rectal abnormal crypt lesions compared to the placebo group (*p*=0.5631)
NCT01239095	FASN	EGCG	Phase I	Terminated; No Study Results Posted
NCT02891538	FASN	EGCG	Early Phase I	Active, not recruiting; No Study Results Posted
NCT01360320	FASN	EGCG	Phase II	Non-significant reduction in adenoma incidence (RR=0.883, *p*=0.1169)
NCT02321969	FASN	ECCG	Not Applicable	Significant reduction in metachronous adenomas (RR=0.56, 95%CI 0.34-0.92)
NCT02980029	FASN	TVB-2640	Phase I	Terminated; No Study Results Posted

**Table 3** summarizes preclinical therapeutic compounds targeting lipid metabolism in CRC.

**Table 3:** The preclinical research on targeting lipid metabolism in CRC.

Main target	Compound	Mechanism	References
FASN	Cerulein	Inhibits Akt phosphorylation; combined with oxaliplatin induces p53-p21 pathway and caspase-3 apoptosis	(193, 194)
	EGCG	Reduce the expression of p‐Akt and p‐ERK1/2; decreases the ATP level	(195)
	Luteolin	Upregulates miR-384 to suppress PTN; induces G1/G2 arrest and DR5-mediated apoptosis	(198).
	TVB-3664	Inhibits lipid synthesis; enhances anti-proliferation with CD36 inhibitors; modulates Akt/Erk signaling	(25)
ACLy	SB204990	Prodrug hydrolyzed to active inhibitor SB201076; reduces acetyl-CoA production and plasma cholesterol/triglycerides	(201)
ACCA	TOFA	Allosterically inhibits ACCA to block malonyl-CoA; induces G0/G1 arrest and caspase-3 apoptosis	(202)
SREBP	Betulinic Acid	Inhibits SREBP transcription factors; downregulates lipogenesis genes	(203)
CPT1	Etomoxir	Blocks fatty acid beta-oxidation	(204)
	DHP-B	Binds to Cys96 of CPT1A, blocks FAO, and disrupts the mitochondrial CPT1A-VDAC1 interaction	(77)

### 5.2 Potential of combination therapy

The combination of lipid metabolism inhibitors and chemotherapy is a promising therapeutic approach. Oxaliplatin and 5-fluorouracil are mainstream chemotherapy drugs for the treatment of CRC, and reducing resistance to them is crucial for CRC treatment (8). In xenograft mouse models, the combination of Cerulenin and oxaliplatin significantly inhibits CRC progression and reduces the dosages of both drugs, thereby extending chemotherapy duration (205); EGCG can target CRC tumor stem cells and enhance their sensitivity to 5-fluorouracil chemotherapy (206).ACLy can induce resistance in CRC cells to the active metabolite of irinotecan, SN38, but the combination of ACLy and AKT inhibitors can restore chemotherapy sensitivity in tumor cells (207). In organoids derived from CRC patients resistant to cetuximab, the FABP4 inhibitor BMS309403 can restore tumor cells’ sensitivity to cetuximab (134). In preclinical studies, the SCD1 inhibitor MF-438, when combined with 5-fluorouracil, can reduce the 5-FU dose by half while maintaining the same efficacy (16). Moreover, targeting lipid metabolism can synergize with immunotherapy: for example, SIRT is a gene involved in the conversion of glucose-lipid metabolism in cells. In CRC, SIRT1 promotes the secretion of CX3CL1, which enhances Treg cell infiltration. CX3CR1 inhibitors can suppress CRC proliferation while enhancing the inhibitory effect of PD-1 antibodies on the tumor (208). **Table 4** outlines the effects of combining lipid metabolism inhibitors with chemotherapy, immunotherapy, or targeted drugs

**Table 4:** The combination therapy in CRC.

Main target	Treatment	Combination	Key Effect	References
FASN	Cerulenin	Oxaliplati	Synergistically inhibits CRC progression in xenografts, enables dose reduction	(205)
FASN	EGCG	5-FU	Sensitizes CRC stem cells to 5-FU	(206)
ACLy	ACLY inhibitor	Irinotecan (SN38)	Overcomes SN38 resistance via AKT inhibition	(207)
FABP4	BMS309403	Cetuximab	Restores cetuximab sensitivity in resistant CRC organoids	(134)
SCD1	MF-438	5-FU	Enables 50% 5-FU dose reduction without efficacy loss	(16)
PPAR	Bezafibrate	Anti-PD-1	Boosts T-cell FAO, reduces apoptosis, enhances anti-PD-1 response	(208)

## 6 Conclusions

Lipid metabolism reprogramming plays a crucial role in the progression of CRC. During the development of CRC, the expression of key enzymes involved in processes such as fatty acid synthesis, uptake, and oxidation is often increased. This process not only provides an abundant energy supply for tumor cells but also promotes malignant progression by regulating cell signaling, enhancing anti-apoptotic mechanisms, and increasing cell migration and invasion capabilities. Factors in the TME, such as nutritional status, hypoxic conditions, and immune responses, directly affect the lipid metabolism pathways of tumor cells, thereby promoting tumor invasiveness and resistance (124). Various cell types within the TME, including Tregs, CAAs, and CAFs, undergo lipid metabolism reprogramming through interactions with the tumor, fostering an immune-suppressive microenvironment that promotes metastasis (99). At the same time, the gut microbiota, an important component of the TME, can influence tumor cell metabolic reprogramming by modulating metabolic products and immune responses in the intestine. The lipid metabolism reprogramming of CRC cells also leads to further dysregulation of the gut microbiota, accelerating tumor progression.

The reprogramming of lipid metabolism in CRC leads to changes in specific lipid molecular profiles, making these molecules highly promising diagnostic and prognostic biomarkers. With the rapid development of lipidomics technologies, especially the application of MS-based high-throughput analysis platforms and multivariate statistical methods, the identification and quantification of these lipid markers have become feasible. However, although existing studies have revealed a large number of lipid metabolism-related molecules with clinical potential, their translational application in clinical practice still faces challenges such as insufficient standardization and lack of cross-cohort validation. Therefore, future research should further focus on the mechanistic analysis of lipid markers, the standardization of analysis processes, and clinical validation with multi-center large samples, so as to promote the effective application of lipidomics in the precision diagnosis and treatment of CRC.

Currently, effective treatments for advanced-stage CRC patients are still lacking. Given the central role of lipid metabolism in CRC initiation and progression, many drugs or inhibitors tested in preclinical models target lipid metabolism. Studies have shown that targeting lipid metabolism can inhibit CRC proliferation and migration, as well as reduce chemotherapy resistance (209). Some drugs, such as EGCG and TVB-2640, have entered clinical trials (25, 196). However, challenges remain: First, lipid metabolic pathways are redundant, and CRC cells compensate by activating fatty acid uptake pathways to reduce the effect of FASN inhibitors. Therefore, single-target treatments are often difficult to achieve long-term effects. Second, the spatial heterogeneity of the tumor microenvironment means that tumor cells in different regions may have distinct metabolic characteristics. Lipid metabolic capacity is enhanced in hypoxic regions (99). Furthermore, individual differences in metabolic regulation may also affect the effectiveness of targeted therapies, such as in obese patients, where enhanced lipid metabolism makes them more prone to resistance (210). Therefore, future research should integrate multi-omics technologies, such as spatial metabolomics and single-cell transcriptomics, to analyze the dynamic architecture of metabolic networks, and use artificial intelligence models to predict combination targets, ultimately achieving a leap from mechanistic exploration to precision therapy.

## References

[1] Siegel RL Giaquinto AN Jemal A. Cancer statistics, 2024. CA Cancer J Clin. (2024) 74:12–49. doi: 10.3322/caac.21820, PMID: 38230766

[2] Cao W Qin K Li F Chen W. Socioeconomic inequalities in cancer incidence and mortality: An analysis of GLOBOCAN 2022. Chin Med J (Engl). (2024) 137:1407–13. doi: 10.1097/CM9.0000000000003140, PMID: 38616547 PMC11188912

[3] He M Gu R Huang X Zhao A Tian S Zheng Y. Global burden of colorectal cancer attributable to metabolic risks from 1990 to 2021, with projections of mortality to 2035. Int J Colorectal Dis. (2025) 40:46. doi: 10.1007/s00384-025-04817-w, PMID: 39969585 PMC11839689

[4] Morgan E Arnold M Gini A Lorenzoni V Cabasag CJ Laversanne M. Global burden of colorectal cancer in 2020 and 2040: incidence and mortality estimates from GLOBOCAN. Gut. (2023) 72:338–44. doi: 10.1136/gutjnl-2022-327736, PMID: 36604116

[5] Abakushina EV Gelm YV Pasova IA Bazhin AV. Immunotherapeutic approaches for the treatment of colorectal cancer. Biochem (Mosc). (2019) 84:720–8. doi: 10.1134/S0006297919070046, PMID: 31509724

[6] Liu R Ji Z Wang X Zhu L Xin J Ma L. Regorafenib plus sintilimab as a salvage treatment for microsatellite stable metastatic colorectal cancer: a single-arm, open-label, phase II clinical trial. Nat Commun. (2025) 16:1481. doi: 10.1038/s41467-025-56748-3, PMID: 39929851 PMC11811139

[7] Gu T Qi H Wang J Sun L Su Y Hu H. Identification of T cell dysfunction molecular subtypes and exploration of potential immunotherapy targets in BRAF V600E-mutant colorectal cancer. Discover Oncol. (2025) 16:163. doi: 10.1007/s12672-025-01930-8, PMID: 39934467 PMC11813846

[8] Abedizadeh R Majidi F Khorasani HR Abedi H Sabour D. Colorectal cancer: a comprehensive review of carcinogenesis, diagnosis, and novel strategies for classified treatments. Cancer Metastasis Rev. (2024) 43:729–53. doi: 10.1007/s10555-023-10158-3, PMID: 38112903

[9] Rosati G Aprile G Colombo A Cordio S Giampaglia M Cappetta A. Colorectal cancer heterogeneity and the impact on precision medicine and therapy efficacy. Biomedicines. (2022) 10:1035. doi: 10.3390/biomedicines10051035, PMID: 35625772 PMC9138254

[10] Acha-Sagredo A Andrei P Clayton K Taggart E Antoniotti C Woodman CA. A constitutive interferon-high immunophenotype defines response to immunotherapy in colorectal cancer. Cancer Cell. (2025) 43:292–307.e7. doi: 10.1016/j.ccell.2024.12.008, PMID: 39824178

[11] Wang D Zhu L Liu H Feng X Zhang C Liu B. Altered gut metabolites and metabolic reprogramming involved in the pathogenesis of colitis-associated colorectal cancer and the transition of colon "inflammation to cancer. J Pharm Biomed Analysis. (2025) 253:116553. doi: 10.1016/j.jpba.2024.116553, PMID: 39486392

[12] Schmidt DR Patel R Kirsch DG Lewis CA Vander Heiden MG Locasale JW. Metabolomics in cancer research and emerging applications in clinical oncology. CA Cancer J Clin. (2021) 71:333–58. doi: 10.3322/caac.21670, PMID: 33982817 PMC8298088

[13] Faubert B Solmonson A DeBerardinis RJ. Metabolic reprogramming and cancer progression. Science. (2020) 368:eaaw5473. doi: 10.1126/science.aaw5473, PMID: 32273439 PMC7227780

[14] Sun L Zhang H Gao P. Metabolic reprogramming and epigenetic modifications on the path to cancer. Protein Cell. (2022) 13:877–919. doi: 10.1007/s13238-021-00846-7, PMID: 34050894 PMC9243210

[15] Marcucci F Rumio C. On the role of glycolysis in early tumorigenesis-permissive and executioner effects. Cells. (2023) 12:1124. doi: 10.3390/cells12081124, PMID: 37190033 PMC10137279

[16] Zabielska J Stelmanska E Szrok-Jurga S Kobiela J Czumaj A. Lipids metabolism inhibition antiproliferative synergy with 5-fluorouracil in human colorectal cancer model. Int J Mol Sci. (2025) 26:1186. doi: 10.3390/ijms26031186, PMID: 39940954 PMC11818398

[17] Krishnan ST Winkler D Creek D Anderson D Kirana C Maddern GJ. Staging of colorectal cancer using lipid biomarkers and machine learning. Metabolomics. (2023) 19:84. doi: 10.1007/s11306-023-02049-z, PMID: 37731020 PMC10511619

[18] Ma Y Zhang S Jin Z Shi M. Lipid-mediated regulation of the cancer-immune crosstalk. Pharmacol Res. (2020) 161:105131. doi: 10.1016/j.phrs.2020.105131, PMID: 32810628

[19] Luo X Cheng C Tan Z Li N Tang M Yang L. Emerging roles of lipid metabolism in cancer metastasis. Mol Cancer. (2017) 16:76. doi: 10.1186/s12943-017-0646-3, PMID: 28399876 PMC5387196

[20] Efeyan A Comb WC Sabatini DM. Nutrient-sensing mechanisms and pathways. Nature. (2015) 517:302–10. doi: 10.1038/nature14190, PMID: 25592535 PMC4313349

[21] Gomaraschi M. Role of lipoproteins in the microenvironment of hormone-dependent cancers. Trends Endocrinol Metab. (2020) 31:256–68. doi: 10.1016/j.tem.2019.11.005, PMID: 31837908

[22] Snaebjornsson MT Janaki-Raman S Schulze A. Greasing the wheels of the cancer machine: the role of lipid metabolism in cancer. Cell Metab. (2020) 31:62–76. doi: 10.1016/j.cmet.2019.11.010, PMID: 31813823

[23] Piccinin E Cariello M Moschetta A. Lipid metabolism in colon cancer: Role of Liver X Receptor (LXR) and Stearoyl-CoA Desaturase 1 (SCD1). Mol Aspects Med. (2021) 78:100933. doi: 10.1016/j.mam.2020.100933, PMID: 33218679

[24] Wang Y Qiu X Li Q Qin J Ye L Zhang X. Single-cell and spatial-resolved profiling reveals cancer-associated fibroblast heterogeneity in colorectal cancer metabolic subtypes. J Trans Med. (2025) 23:175. doi: 10.1186/s12967-025-06103-3, PMID: 39934919 PMC11817247

[25] Zaytseva YY Rychahou PG Le AT Scott TL Flight RM Kim JT. Preclinical evaluation of novel fatty acid synthase inhibitors in primary colorectal cancer cells and a patient-derived xenograft model of colorectal cancer. Oncotarget. (2018) 9:24787–800. doi: 10.18632/oncotarget.25361, PMID: 29872506 PMC5973868

[26] Jog E Jainarayanan AK La Ferlita A Chakraborty A Dalwai A Yahya S. Inhibiting *de novo* lipogenesis identifies a therapeutic vulnerability in therapy-resistant colorectal cancer. Redox Biol. (2025) 79:103458. doi: 10.1016/j.redox.2024.103458, PMID: 39705849 PMC11729006

[27] Liu H Wang S Wang J Guo X Song Y Fu K. Energy metabolism in health and diseases. Signal Transduction Targeted Ther. (2025) 10:69. doi: 10.1038/s41392-025-02141-x, PMID: 39966374 PMC11836267

[28] Lam SM Wang Z Li B Shui G. High-coverage lipidomics for functional lipid and pathway analyses. Anal Chim Acta. (2021) 1147:199–210. doi: 10.1016/j.aca.2020.11.024, PMID: 33485579

[29] Schönfeld P Wojtczak L. Short- and medium-chain fatty acids in energy metabolism: the cellular perspective. J Lipid Res. (2016) 57:943–54. doi: 10.1194/jlr.R067629, PMID: 27080715 PMC4878196

[30] Matsushita Y Nakagawa H Koike K. Lipid metabolism in oncology: why it matters, how to research, and how to treat. Cancers (Basel). (2021) 13:474. doi: 10.3390/cancers13030474, PMID: 33530546 PMC7865757

[31] Feng WW Wilkins O Bang S Ung M Li J An J. CD36-mediated metabolic rewiring of breast cancer cells promotes resistance to HER2-targeted therapies. Cell Rep. (2019) 29:3405–20.e5. doi: 10.1016/j.celrep.2019.11.008, PMID: 31825825 PMC6938262

[32] Wang J Wen T Li Z Che X Gong L Jiao Z. CD36 upregulates DEK transcription and promotes cell migration and invasion via GSK-3β/β-catenin-mediated epithelial-to-mesenchymal transition in gastric cancer. Aging (Albany NY). (2020) 13:1883–97. doi: 10.18632/aging.103985, PMID: 33232276 PMC7880392

[33] Kuang H Sun X Liu Y Tang M Wei Y Shi Y. Palmitic acid-induced ferroptosis via CD36 activates ER stress to break calcium-iron balance in colon cancer cells. FEBS J. (2023) 290:3664–87. doi: 10.1111/febs.v290.14, PMID: 36906928

[34] Pascual G Avgustinova A Mejetta S Martín M Castellanos A Attolini CS. Targeting metastasis-initiating cells through the fatty acid receptor CD36. Nature. (2017) 541:41–5. doi: 10.1038/nature20791, PMID: 27974793

[35] Zhang F Xia X Chai R Xu R Xu Q Liu M. Inhibition of USP14 suppresses the formation of foam cell by promoting CD36 degradation. J Cell Mol Med. (2020) 24:3292–302. doi: 10.1111/jcmm.15002, PMID: 31970862 PMC7131911

[36] Ruan C Meng Y Song H. CD36: an emerging therapeutic target for cancer and its molecular mechanisms. J Cancer Res Clin Oncol. (2022) 148:1551–8. doi: 10.1007/s00432-022-03957-8, PMID: 35224665 PMC11801024

[37] Drury J Rychahou PG Kelson CO Geisen ME Wu Y He D. Upregulation of CD36, a fatty acid translocase, promotes colorectal cancer metastasis by increasing MMP28 and decreasing E-cadherin expression. Cancers (Basel). (2022) 14:252. doi: 10.3390/cancers14010252, PMID: 35008415 PMC8750155

[38] Drury J Rychahou PG He D Jafari N Wang C Lee EY. Inhibition of fatty acid synthase upregulates expression of CD36 to sustain proliferation of colorectal cancer cells. Front Oncol. (2020) 10:1185. doi: 10.3389/fonc.2020.01185, PMID: 32850342 PMC7411002

[39] Zhang X Yao J Shi H Gao B Zhang L. LncRNA TINCR/microRNA-107/CD36 regulates cell proliferation and apoptosis in colorectal cancer via PPAR signaling pathway based on bioinformatics analysis. Biol Chem. (2019) 400:663–75. doi: 10.1515/hsz-2018-0236, PMID: 30521471

[40] Koundouros N Poulogiannis G. Reprogramming of fatty acid metabolism in cancer. Br J Cancer. (2020) 122:4–22. doi: 10.1038/s41416-019-0650-z, PMID: 31819192 PMC6964678

[41] Binienda A Machelak W Zielińska M Fichna J. Free fatty acid receptors type 2 and 4 mediate the anticancer effects of fatty acids in colorectal cancer - *in vitro* and *in vivo* studies. Biochim Biophys Acta (BBA) - Mol Basis Disease. (2025) 1871:167708. doi: 10.1016/j.bbadis.2025.167708, PMID: 39922546

[42] Zhang M Di Martino JS Bowman RL Campbell NR Baksh SC Simon-Vermot T. Adipocyte-derived lipids mediate melanoma progression via FATP proteins. Cancer Discov. (2018) 8:1006–25. doi: 10.1158/2159-8290.CD-17-1371, PMID: 29903879 PMC6192670

[43] Geng QS Yang MJ Li LF Shen ZB Wang LH Zheng YY. Over-expression and prognostic significance of FATP5, as a new biomarker, in colorectal carcinoma. Front Mol Biosci. (2021) 8:770624. doi: 10.3389/fmolb.2021.770624, PMID: 35155561 PMC8829069

[44] Dharmarajan S Newberry EP Montenegro G Nalbantoglu I Davis VR Clanahan MJ. Liver fatty acid-binding protein (L-Fabp) modifies intestinal fatty acid composition and adenoma formation in ApcMin/+ mice. Cancer Prev Res (Phila). (2013) 6:1026–37. doi: 10.1158/1940-6207.CAPR-13-0120, PMID: 23921281 PMC3791217

[45] Kawaguchi K Senga S Kubota C Kawamura Y Ke Y Fujii H. High expression of Fatty Acid-Binding Protein 5 promotes cell growth and metastatic potential of colorectal cancer cells. FEBS Open Bio. (2016) 6:190–9. doi: 10.1002/2211-5463.12031, PMID: 27047747 PMC4794781

[46] Tian W Zhang W Zhang Y Zhu T Hua Y Li H. FABP4 promotes invasion and metastasis of colon cancer by regulating fatty acid transport. Cancer Cell Int. (2020) 20:512. doi: 10.1186/s12935-020-01582-4, PMID: 33088219 PMC7574203

[47] Kim SH Pyo JS Son BK Oh IH Min KW. Clinicopathological significance and prognostic implication of nuclear fatty acid-binding protein 4 expression in colorectal cancer. Pathol Res Pract. (2023) 249:154722. doi: 10.1016/j.prp.2023.154722, PMID: 37591068

[48] Pakiet A Sikora K Kobiela J Rostkowska O Mika A Sledzinski T. Alterations in complex lipids in tumor tissue of patients with colorectal cancer. Lipids Health Dis. (2021) 20:85. doi: 10.1186/s12944-021-01512-x, PMID: 34348720 PMC8340484

[49] Wen J Min X Shen M Hua Q Han Y Zhao L. ACLY facilitates colon cancer cell metastasis by CTNNB1. J Exp Clin Cancer Res. (2019) 38:401. doi: 10.1186/s13046-019-1391-9, PMID: 31511060 PMC6740040

[50] Zhang M Peng R Wang H Yang Z Zhang H Zhang Y. Nanog mediated by FAO/ACLY signaling induces cellular dormancy in colorectal cancer cells. Cell Death Dis. (2022) 13:159. doi: 10.1038/s41419-022-04606-1, PMID: 35177584 PMC8854412

[51] Yang Y Luo D Shao Y Shan Z Liu Q Weng J. circCAPRIN1 interacts with STAT2 to promote tumor progression and lipid synthesis via upregulating ACC1 expression in colorectal cancer. Cancer Commun (Lond). (2023) 43:100–22. doi: 10.1002/cac2.12380, PMID: 36328987 PMC9859733

[52] Li S Lu CW Diem EC Li W Guderian M Lindenberg M. Acetyl-CoA-Carboxylase 1-mediated *de novo* fatty acid synthesis sustains Lgr5(+) intestinal stem cell function. Nat Commun. (2022) 13:3998. doi: 10.1038/s41467-022-31725-2, PMID: 35810180 PMC9271096

[53] Röhrig F Schulze A. The multifaceted roles of fatty acid synthesis in cancer. Nat Rev Cancer. (2016) 16:732–49. doi: 10.1038/nrc.2016.89, PMID: 27658529

[54] Fhu CW Ali A. Fatty acid synthase: an emerging target in cancer. Molecules. (2020) 25:3935. doi: 10.3390/molecules25173935, PMID: 32872164 PMC7504791

[55] Ecker J Benedetti E Kindt ASD Höring M Perl M Machmüller AC. The colorectal cancer lipidome: identification of a robust tumor-specific lipid species signature. Gastroenterology. (2021) 161:910–23.e19. doi: 10.1053/j.gastro.2021.05.009, PMID: 34000281

[56] Lu T Sun L Wang Z Zhang Y He Z Xu C. Fatty acid synthase enhances colorectal cancer cell proliferation and metastasis via regulating AMPK/mTOR pathway. Onco Targets Ther. (2019) 12:3339–47. doi: 10.2147/OTT.S199369, PMID: 31118685 PMC6504633

[57] Zhao Y Liu MJ Zhang L Yang Q Sun QH Guo JR. High mobility group A1 (HMGA1) promotes the tumorigenesis of colorectal cancer by increasing lipid synthesis. Nat Commun. (2024) 15:9909. doi: 10.1038/s41467-024-54400-0, PMID: 39548107 PMC11568219

[58] Sen U Coleman C Sen T. Stearoyl coenzyme A desaturase-1: multitasker in cancer, metabolism, and ferroptosis. Trends Cancer. (2023) 9:480–9. doi: 10.1016/j.trecan.2023.03.003, PMID: 37029018

[59] Wu T Wan J Qu X Xia K Wang F Zhang Z. Nodal promotes colorectal cancer survival and metastasis through regulating SCD1-mediated ferroptosis resistance. Cell Death Dis. (2023) 14:229. doi: 10.1038/s41419-023-05756-6, PMID: 37002201 PMC10066180

[60] Che G Wang W Wang J He C Yin J Chen Z. Sulfotransferase SULT2B1 facilitates colon cancer metastasis by promoting SCD1-mediated lipid metabolism. Clin Transl Med. (2024) 14:e1587. doi: 10.1002/ctm2.v14.2, PMID: 38372484 PMC10875708

[61] Nagarajan SR Butler LM Hoy AJ. The diversity and breadth of cancer cell fatty acid metabolism. Cancer Metab. (2021) 9:2. doi: 10.1186/s40170-020-00237-2, PMID: 33413672 PMC7791669

[62] Menendez JA Lupu R. Fatty acid synthase and the lipogenic phenotype in cancer pathogenesis. Nat Rev Cancer. (2007) 7:763–77. doi: 10.1038/nrc2222, PMID: 17882277

[63] Olzmann JA Carvalho P. Dynamics and functions of lipid droplets. Nat Rev Mol Cell Biol. (2019) 20:137–55. doi: 10.1038/s41580-018-0085-z, PMID: 30523332 PMC6746329

[64] Petan T Jarc E Jusović M. Lipid droplets in cancer: guardians of fat in a stressful world. Molecules. (2018) 23:1941. doi: 10.3390/molecules23081941, PMID: 30081476 PMC6222695

[65] Butler LM Perone Y Dehairs J Lupien LE de Laat V Talebi A. Lipids and cancer: Emerging roles in pathogenesis, diagnosis and therapeutic intervention. Adv Drug Delivery Rev. (2020) 159:245–93. doi: 10.1016/j.addr.2020.07.013, PMID: 32711004 PMC7736102

[66] Munir R Lisec J Jaeger C Zaidi N. Abundance, fatty acid composition and saturation index of neutral lipids in colorectal cancer cell lines. Acta Biochim Pol. (2021) 68:115–8. doi: 10.18388/abp.2020_5465, PMID: 33595227

[67] Tirinato L Liberale C Di Franco S Candeloro P Benfante A La Rocca R. Lipid droplets: a new player in colorectal cancer stem cells unveiled by spectroscopic imaging. Stem Cells. (2015) 33:35–44. doi: 10.1002/stem.1837, PMID: 25186497 PMC4311668

[68] Liu H Du J Chao S Li S Cai H Zhang H. Fusobacterium nucleatum promotes colorectal cancer cell to acquire stem cell-like features by manipulating lipid droplet-mediated numb degradation. Adv Sci (Weinh). (2022) 9:e2105222. doi: 10.1002/advs.202105222, PMID: 35170250 PMC9035998

[69] Pillai SR Damaghi M Marunaka Y Spugnini EP Fais S Gillies RJ. Causes, consequences, and therapy of tumors acidosis. Cancer Metastasis Rev. (2019) 38:205–22. doi: 10.1007/s10555-019-09792-7, PMID: 30911978 PMC6625890

[70] Khacho M Tarabay M Patten D Khacho P MacLaurin JG Guadagno J. Acidosis overrides oxygen deprivation to maintain mitochondrial function and cell survival. Nat Commun. (2014) 5:3550. doi: 10.1038/ncomms4550, PMID: 24686499 PMC3988820

[71] Mailler E Guardia CM Bai X Jarnik M Williamson CD Li Y. The autophagy protein ATG9A enables lipid mobilization from lipid droplets. Nat Commun. (2021) 12:6750. doi: 10.1038/s41467-021-26999-x, PMID: 34799570 PMC8605025

[72] Liu X Sun X Mu W Li Y Bu W Yang T. Autophagic flux-lipid droplet biogenesis cascade sustains mitochondrial fitness in colorectal cancer cells adapted to acidosis. Cell Death Discovery. (2025) 11:21. doi: 10.1038/s41420-025-02301-6, PMID: 39856069 PMC11761495

[73] Martinez-Outschoorn UE Peiris-Pagés M Pestell RG Sotgia F Lisanti MP. Cancer metabolism: a therapeutic perspective. Nat Rev Clin Oncol. (2017) 14:11–31. doi: 10.1038/nrclinonc.2016.60, PMID: 27141887

[74] Ma Y Temkin SM Hawkridge AM Guo C Wang W Wang XY. Fatty acid oxidation: An emerging facet of metabolic transformation in cancer. Cancer Lett. (2018) 435:92–100. doi: 10.1016/j.canlet.2018.08.006, PMID: 30102953 PMC6240910

[75] Bian X Liu R Meng Y Xing D Xu D Lu Z. Lipid metabolism and cancer. J Exp Med. (2021) 218:e20201606. doi: 10.1084/jem.20201606, PMID: 33601415 PMC7754673

[76] Zou P Chen C Wu X. Identification of fatty acid oxidation-related subtypes by integrated analysis of bulk- and single-cell transcriptome profiling in colorectal cancer. J Gastrointest Oncol. (2024) 15:147–63. doi: 10.21037/jgo-23-833, PMID: 38482228 PMC10932680

[77] Hu A Wang H Xu Q Pan Y Jiang Z Li S. A novel CPT1A covalent inhibitor modulates fatty acid oxidation and CPT1A-VDAC1 axis with therapeutic potential for colorectal cancer. Redox Biol. (2023) 68:102959. doi: 10.1016/j.redox.2023.102959, PMID: 37977042 PMC10692921

[78] Perše M. Oxidative stress in the pathogenesis of colorectal cancer: Cause or consequence? BioMed Res Int. (2013) 2013:725710. doi: 10.1155/2013/725710, PMID: 23762854 PMC3666330

[79] Lei L Yang J Zhang J Zhang G. The lipid peroxidation product EKODE exacerbates colonic inflammation and colon tumorigenesis. Redox Biol. (2021) 42:101880. doi: 10.1016/j.redox.2021.101880, PMID: 33541845 PMC8113040

[80] Yi J Zhu J Wu J Thompson CB Jiang X. Oncogenic activation of PI3K-AKT-mTOR signaling suppresses ferroptosis via SREBP-mediated lipogenesis. Proc Natl Acad Sci U S A. (2020) 117:31189–97. doi: 10.1073/pnas.2017152117, PMID: 33229547 PMC7733797

[81] Tang H Zhang Y Zhao D Guo M Yuan X Wang X. Unlocking the lipid code: SREBPs as key drivers in gastrointestinal tumour metabolism. Lipids Health Dis. (2025) 24:190. doi: 10.1186/s12944-025-02612-8, PMID: 40413517 PMC12103804

[82] Wen YA Xiong X Zaytseva YY Napier DL Vallee E Li AT. Downregulation of SREBP inhibits tumor growth and initiation by altering cellular metabolism in colon cancer. Cell Death Dis. (2018) 9:265. doi: 10.1038/s41419-018-0330-6, PMID: 29449559 PMC5833501

[83] Wang H Chen Y Liu Y Li Q Luo J Wang L. The lncRNA ZFAS1 regulates lipogenesis in colorectal cancer by binding polyadenylate-binding protein 2 to stabilize SREBP1 mRNA. Mol Ther Nucleic Acids. (2022) 27:363–74. doi: 10.1016/j.omtn.2021.12.010, PMID: 35036050 PMC8728310

[84] Liu YL Xiang Z Zhang BY Zou YW Chen GL Yin L. APOA5 alleviates reactive oxygen species to promote oxaliplatin resistance in PIK3CA-mutated colorectal cancer. Aging (Albany NY). (2024) 16:9410–36. doi: 10.18632/aging.205872, PMID: 38848145 PMC11210231

[85] Xu M Jiang SY Tang S Zhu M Hu Y Li J. Nuclear SREBP2 condensates regulate the transcriptional activation of lipogenic genes and cholesterol homeostasis. Nat Metab. (2025) 7:1034–51. doi: 10.1038/s42255-025-01291-0, PMID: 40394324

[86] Zhang KL Zhu WW Wang SH Gao C Pan JJ Du ZG. Organ-specific cholesterol metabolic aberration fuels liver metastasis of colorectal cancer. Theranostics. (2021) 11:6560–72. doi: 10.7150/thno.55609, PMID: 33995676 PMC8120208

[87] Muta Y Linares JF Martinez-Ordoñez A Duran A Cid-Diaz T Kinoshita H. Enhanced SREBP2-driven cholesterol biosynthesis by PKCλ/ι deficiency in intestinal epithelial cells promotes aggressive serrated tumorigenesis. Nat Commun. (2023) 14:8075. doi: 10.1038/s41467-023-43690-5, PMID: 38092754 PMC10719313

[88] Buñay J Fouache A Trousson A de Joussineau C Bouchareb E Zhu Z. Screening for liver X receptor modulators: Where are we and for what use? Br J Pharmacol. (2021) 178:3277–93. doi: 10.1111/bph.15286, PMID: 33080050

[89] Duval C Touche V Tailleux A Fruchart JC Fievet C Clavey V. Niemann-Pick C1 like 1 gene expression is down-regulated by LXR activators in the intestine. Biochem Biophys Res Commun. (2006) 340:1259–63. doi: 10.1016/j.bbrc.2005.12.137, PMID: 16414355

[90] Repa JJ Berge KE Pomajzl C Richardson JA Hobbs H Mangelsdorf DJ. Regulation of ATP-binding cassette sterol transporters ABCG5 and ABCG8 by the liver X receptors α and β. J Biol Chem. (2002) 277:18793–800. doi: 10.1074/jbc.M109927200, PMID: 11901146

[91] Sharma B Gupta V Dahiya D Kumar H Vaiphei K Agnihotri N. Clinical relevance of cholesterol homeostasis genes in colorectal cancer. Biochim Biophys Acta - Mol Cell Biol Lipids. (2019) 1864:1314–27. doi: 10.1016/j.bbalip.2019.06.008, PMID: 31202724

[92] Uno S Endo K Jeong Y Kawana K Miyachi H Hashimoto Y. Suppression of beta-catenin signaling by liver X receptor ligands. Biochem Pharmacol. (2009) 77:186–95. doi: 10.1016/j.bcp.2008.10.007, PMID: 18983830

[93] Das S Parigi SM Luo X Fransson J Kern BC Okhovat A. Liver X receptor unlinks intestinal regeneration and tumorigenesis. Nature. (2025) 637:1198–206. doi: 10.1038/s41586-024-08247-6, PMID: 39567700 PMC11779645

[94] Luo Y Xie C Brocker CN Fan J Wu X Feng L. Intestinal PPARα Protects against colon carcinogenesis via regulation of methyltransferases DNMT1 and PRMT6. Gastroenterology. (2019) 157:744–59.e4. doi: 10.1053/j.gastro.2019.05.057, PMID: 31154022 PMC7388731

[95] Rolver MG Holland LKK Ponniah M Prasad NS Yao J Schnipper J. Chronic acidosis rewires cancer cell metabolism through PPARα signaling. Int J Cancer. (2023) 152:1668–84. doi: 10.1002/ijc.v152.8 PMC10108231 36533672

[96] Mana MD Hussey AM Tzouanas CN Imada S Barrera Millan Y Bahceci D. High-fat diet-activated fatty acid oxidation mediates intestinal stemness and tumorigenicity. Cell Rep. (2021) 35:109212. doi: 10.1016/j.celrep.2021.109212, PMID: 34107251 PMC8258630

[97] Kim JT Li C Weiss HL Zhou Y Liu C Wang Q. Regulation of ketogenic enzyme HMGCS2 by wnt/β-catenin/PPARγ Pathway in intestinal cells. Cells. (2019) 8:1106. doi: 10.3390/cells8091106, PMID: 31546785 PMC6770209

[98] Mokhtari K Mahdevar M Hajipour M Esmaeili M Peymani M Mirzaei S. Title: Involvement of unsaturated fatty acid biosynthesis in CRC progression based on *in vitro* and in silico studies. BioMed Pharmacother. (2022) 153:113338. doi: 10.1016/j.biopha.2022.113338, PMID: 35779418

[99] Nenkov M Ma Y Gaßler N Chen Y. Metabolic reprogramming of colorectal cancer cells and the microenvironment: implication for therapy. Int J Mol Sci. (2021) 22:6262. doi: 10.3390/ijms22126262, PMID: 34200820 PMC8230539

[100] Krauß D Fari O Sibilia M. Lipid metabolism interplay in CRC-an update. Metabolites. (2022) 12:213. doi: 10.3390/metabo12030213, PMID: 35323656 PMC8951276

[101] Long Y Shi H He Y Qi X. Analyzing the impact of metabolism on immune cells in tumor microenvironment to promote the development of immunotherapy. Front Immunol. (2023) 14:1307228. doi: 10.3389/fimmu.2023.1307228, PMID: 38264667 PMC10804850

[102] Liu L Mo M Chen X Chao D Zhang Y Chen X. Targeting inhibition of prognosis-related lipid metabolism genes including CYP19A1 enhances immunotherapeutic response in colon cancer. J Exp Clin Cancer Res. (2023) 42:85. doi: 10.1186/s13046-023-02647-8, PMID: 37055842 PMC10100168

[103] Pan Y Tian T Park CO Lofftus SY Mei S Liu X. Survival of tissue-resident memory T cells requires exogenous lipid uptake and metabolism. Nature. (2017) 543:252–6. doi: 10.1038/nature21379, PMID: 28219080 PMC5509051

[104] Yost KE Satpathy AT Wells DK Qi Y Wang C Kageyama R. Clonal replacement of tumor-specific T cells following PD-1 blockade. Nat Med. (2019) 25:1251–9. doi: 10.1038/s41591-019-0522-3, PMID: 31359002 PMC6689255

[105] Corn KC Windham MA Rafat M. Lipids in the tumor microenvironment: From cancer progression to treatment. Prog Lipid Res. (2020) 80:101055. doi: 10.1016/j.plipres.2020.101055, PMID: 32791170 PMC7674189

[106] He S Cai T Yuan J Zheng X Yang W. Lipid Metabolism in Tumor-Infiltrating T Cells. In: Li Y, editor. Lipid Metabolism in Tumor Immunity. Springer Singapore, Singapore (2021). p. 149–67. 10.1007/978-981-33-6785-2_10 33740249

[107] Michalek RD Gerriets VA Jacobs SR Macintyre AN MacIver NJ Mason EF. Cutting edge: distinct glycolytic and lipid oxidative metabolic programs are essential for effector and regulatory CD4+ T cell subsets. J Immunol. (2011) 186:3299–303. doi: 10.4049/jimmunol.1003613, PMID: 21317389 PMC3198034

[108] Sugiura A Rathmell JC. Metabolic barriers to T cell function in tumors. J Immunol. (2018) 200:400–7. doi: 10.4049/jimmunol.1701041, PMID: 29311381 PMC5777533

[109] Nelson ER Wardell SE Jasper JS Park S Suchindran S Howe MK. 27-Hydroxycholesterol links hypercholesterolemia and breast cancer pathophysiology. Science. (2013) 342:1094–8. doi: 10.1126/science.1241908, PMID: 24288332 PMC3899689

[110] Xu S Chaudhary O Rodríguez-Morales P Sun X Chen D Zappasodi R. Uptake of oxidized lipids by the scavenger receptor CD36 promotes lipid peroxidation and dysfunction in CD8(+) T cells in tumors. Immunity. (2021) 54:1561–77.e7. doi: 10.1016/j.immuni.2021.05.003, PMID: 34102100 PMC9273026

[111] Fernandez-Marcos PJ Auwerx J. Regulation of PGC-1α, a nodal regulator of mitochondrial biogenesis. Am J Clin Nutr. (2011) 93:884s–90. doi: 10.3945/ajcn.110.001917, PMID: 21289221 PMC3057551

[112] He S Cai T Yuan J Zheng X Yang W. Lipid metabolism in tumor-infiltrating T cells. Adv Exp Med Biol. (2021) 1316:149–67. doi: 10.1007/978-981-33-6785-2_10, PMID: 33740249

[113] Wang H Franco F Tsui YC Xie X Trefny MP Zappasodi R. CD36-mediated metabolic adaptation supports regulatory T cell survival and function in tumors. Nat Immunol. (2020) 21:298–308. doi: 10.1038/s41590-019-0589-5, PMID: 32066953 PMC7043937

[114] Miska J Lee-Chang C Rashidi A Muroski ME Chang AL Lopez-Rosas A. HIF-1α Is a metabolic switch between glycolytic-driven migration and oxidative phosphorylation-driven immunosuppression of tregs in glioblastoma. Cell Rep. (2019) 27:226–37.e4. doi: 10.1016/j.celrep.2019.03.029, PMID: 30943404 PMC6461402

[115] Soundararajan R Maurin MM Rodriguez-Silva J Upadhyay G Alden AJ Gowda SGB. Integration of lipidomics with targeted, single cell, and spatial transcriptomics defines an unresolved pro-inflammatory state in colon cancer. Gut. (2025) 74:586–602. doi: 10.1136/gutjnl-2024-332535, PMID: 39658263 PMC11885055

[116] Xiang Y Miao H. Lipid metabolism in tumor-associated macrophages. Adv Exp Med Biol. (2021) 1316:87–101. doi: 10.1007/978-981-33-6785-2_6, PMID: 33740245

[117] Kelly B O'Neill LA. Metabolic reprogramming in macrophages and dendritic cells in innate immunity. Cell Res. (2015) 25:771–84. doi: 10.1038/cr.2015.68, PMID: 26045163 PMC4493277

[118] O'Neill LA Pearce EJ. Immunometabolism governs dendritic cell and macrophage function. J Exp Med. (2016) 213:15–23. doi: 10.1084/jem.20151570, PMID: 26694970 PMC4710204

[119] Chen D Zhang X Li Z Zhu B. Metabolic regulatory crosstalk between tumor microenvironment and tumor-associated macrophages. Theranostics. (2021) 11:1016–30. doi: 10.7150/thno.51777, PMID: 33391518 PMC7738889

[120] Yang P Qin H Li Y Xiao A Zheng E Zeng H. CD36-mediated metabolic crosstalk between tumor cells and macrophages affects liver metastasis. Nat Commun. (2022) 13:5782. doi: 10.1038/s41467-022-33349-y, PMID: 36184646 PMC9527239

[121] Wu H Han Y Rodriguez Sillke Y Deng H Siddiqui S Treese C. Lipid droplet-dependent fatty acid metabolism controls the immune suppressive phenotype of tumor-associated macrophages. EMBO Mol Med. (2019) 11:e10698. doi: 10.15252/emmm.201910698, PMID: 31602788 PMC6835560

[122] Ji T Fu H Wang L Chen J Tian S Wang G. Single-cell RNA profiling reveals classification and characteristics of mononuclear phagocytes in colorectal cancer. PloS Genet. (2024) 20:e1011176. doi: 10.1371/journal.pgen.1011176, PMID: 38408082 PMC10919852

[123] O'Shea D Cawood TJ O'Farrelly C Lynch L. Natural killer cells in obesity: impaired function and increased susceptibility to the effects of cigarette smoke. PloS One. (2010) 5:e8660. doi: 10.1371/journal.pone.0008660, PMID: 20107494 PMC2801590

[124] Jin HR Wang J Wang ZJ Xi MJ Xia BH Deng K. Lipid metabolic reprogramming in tumor microenvironment: from mechanisms to therapeutics. J Hematol Oncol. (2023) 16:103. doi: 10.1186/s13045-023-01498-2, PMID: 37700339 PMC10498649

[125] Niavarani SR Lawson C Bakos O Boudaud M Batenchuk C Rouleau S. Lipid accumulation impairs natural killer cell cytotoxicity and tumor control in the postoperative period. BMC Cancer. (2019) 19:823. doi: 10.1186/s12885-019-6045-y, PMID: 31429730 PMC6701111

[126] Xie S Cai Y Chen D Xiang Y Cai W Mao J. Single-cell transcriptome analysis reveals heterogeneity and convergence of the tumor microenvironment in colorectal cancer. Front Immunol. (2022) 13:1003419. doi: 10.3389/fimmu.2022.1003419, PMID: 36685571 PMC9845924

[127] Veglia F Tyurin VA Mohammadyani D Blasi M Duperret EK Donthireddy L. Lipid bodies containing oxidatively truncated lipids block antigen cross-presentation by dendritic cells in cancer. Nat Commun. (2017) 8:2122. doi: 10.1038/s41467-017-02186-9, PMID: 29242535 PMC5730553

[128] Herber DL Cao W Nefedova Y Novitskiy SV Nagaraj S Tyurin VA. Lipid accumulation and dendritic cell dysfunction in cancer. Nat Med. (2010) 16:880–6. doi: 10.1038/nm.2172, PMID: 20622859 PMC2917488

[129] Tabuso M Homer-Vanniasinkam S Adya R Arasaradnam RP. Role of tissue microenvironment resident adipocytes in colon cancer. World J Gastroenterol. (2017) 23:5829–35. doi: 10.3748/wjg.v23.i32.5829, PMID: 28932075 PMC5583568

[130] Olszańska J Pietraszek-Gremplewicz K Domagalski M Nowak D. Mutual impact of adipocytes and colorectal cancer cells growing in co-culture conditions. Cell Commun Signal. (2023) 21:130. doi: 10.1186/s12964-023-01155-8, PMID: 37316878 PMC10265888

[131] Song Y Na H Lee SE Kim YM Moon J Nam TW. Dysfunctional adipocytes promote tumor progression through YAP/TAZ-dependent cancer-associated adipocyte transformation. Nat Commun. (2024) 15:4052. doi: 10.1038/s41467-024-48179-3, PMID: 38744820 PMC11094189

[132] Mukherjee A Bilecz AJ Lengyel E. The adipocyte microenvironment and cancer. Cancer Metastasis Rev. (2022) 41:575–87. doi: 10.1007/s10555-022-10059-x, PMID: 35941408

[133] Wen YA Xing X Harris JW Zaytseva YY Mitov MI Napier DL. Adipocytes activate mitochondrial fatty acid oxidation and autophagy to promote tumor growth in colon cancer. Cell Death Dis. (2017) 8:e2593. doi: 10.1038/cddis.2017.21, PMID: 28151470 PMC5386470

[134] Cheng YC Chen MY Yadav VK Pikatan NW Fong IH Kuo KT. Targeting FABP4/UCP2 axis to overcome cetuximab resistance in obesity-driven CRC with drug-tolerant persister cells. Transl Oncol. (2025) 53:102274. doi: 10.1016/j.tranon.2025.102274, PMID: 39823981 PMC11787020

[135] Di W Zhang W Zhu B Li X Tang Q Zhou Y. Colorectal cancer prompted adipose tissue browning and cancer cachexia through transferring exosomal miR-146b-5p. J Cell Physiol. (2021) 236:5399–410. doi: 10.1002/jcp.v236.7, PMID: 33368224

[136] Ebadi M Field CJ Lehner R Mazurak VC. Chemotherapy diminishes lipid storage capacity of adipose tissue in a preclinical model of colon cancer. Lipids Health Dis. (2017) 16:247. doi: 10.1186/s12944-017-0638-8, PMID: 29258509 PMC5735884

[137] Murphy RA Wilke MS Perrine M Pawlowicz M Mourtzakis M Lieffers JR. Loss of adipose tissue and plasma phospholipids: relationship to survival in advanced cancer patients. Clin Nutr. (2010) 29:482–7. doi: 10.1016/j.clnu.2009.11.006, PMID: 19959263

[138] Chen X Song E. Turning foes to friends: targeting cancer-associated fibroblasts. Nat Rev Drug Discov. (2019) 18:99–115. doi: 10.1038/s41573-018-0004-1, PMID: 30470818

[139] Kamali Zonouzi S Pezeshki PS Razi S Rezaei N. Cancer-associated fibroblasts in colorectal cancer. Clin Transl Oncol. (2022) 24:757–69. doi: 10.1007/s12094-021-02734-2, PMID: 34839457

[140] Gong J Lin Y Zhang H Liu C Cheng Z Yang X. Reprogramming of lipid metabolism in cancer-associated fibroblasts potentiates migration of colorectal cancer cells. Cell Death Dis. (2020) 11:267. doi: 10.1038/s41419-020-2434-z, PMID: 32327627 PMC7181758

[141] Zhang C Wang XY Zhang P He TC Han JH Zhang R. Cancer-derived exosomal HSPC111 promotes colorectal cancer liver metastasis by reprogramming lipid metabolism in cancer-associated fibroblasts. Cell Death Dis. (2022) 13:57. doi: 10.1038/s41419-022-04506-4, PMID: 35027547 PMC8758774

[142] Peng Z Ye M Ding H Feng Z Hu K. Spatial transcriptomics atlas reveals the crosstalk between cancer-associated fibroblasts and tumor microenvironment components in colorectal cancer. J Transl Med. (2022) 20:302. doi: 10.1186/s12967-022-03510-8, PMID: 35794563 PMC9258101

[143] Dagher Z Ruderman N Tornheim K Ido Y. Acute regulation of fatty acid oxidation and amp-activated protein kinase in human umbilical vein endothelial cells. Circ Res. (2001) 88:1276–82. doi: 10.1161/hh1201.092998, PMID: 11420304

[144] Harjes U Bridges E Gharpure KM Roxanis I Sheldon H Miranda F. Antiangiogenic and tumour inhibitory effects of downregulating tumour endothelial FABP4. Oncogene. (2017) 36:912–21. doi: 10.1038/onc.2016.256, PMID: 27568980 PMC5318662

[145] Hagberg CE Falkevall A Wang X Larsson E Huusko J Nilsson I. Vascular endothelial growth factor B controls endothelial fatty acid uptake. Nature. (2010) 464:917–21. doi: 10.1038/nature08945, PMID: 20228789

[146] Zaytseva YY Elliott VA Rychahou P Mustain WC Kim JT Valentino J. Cancer cell-associated fatty acid synthase activates endothelial cells and promotes angiogenesis in colorectal cancer. Carcinogenesis. (2014) 35:1341–51. doi: 10.1093/carcin/bgu042, PMID: 24510238 PMC4043242

[147] Hussain A Qazi AK Mupparapu N Guru SK Kumar A Sharma PR. Modulation of glycolysis and lipogenesis by novel PI3K selective molecule represses tumor angiogenesis and decreases colorectal cancer growth. Cancer Lett. (2016) 374:250–60. doi: 10.1016/j.canlet.2016.02.030, PMID: 26921131

[148] Xiao G Zheng Y Chen H Luo M Yang C Ren D. Single-cell transcriptome analysis reveals immunosuppressive landscape in overweight and obese colorectal cancer. J Transl Med. (2024) 22:134. doi: 10.1186/s12967-024-04921-5, PMID: 38311726 PMC10838453

[149] Scott AJ Alexander JL Merrifield CA Cunningham D Jobin C Brown R. International Cancer Microbiome Consortium consensus statement on the role of the human microbiome in carcinogenesis. Gut. (2019) 68:1624–32. doi: 10.1136/gutjnl-2019-318556, PMID: 31092590 PMC6709773

[150] Hanus M Parada-Venegas D Landskron G Wielandt AM Hurtado C Alvarez K. Immune system, microbiota, and microbial metabolites: the unresolved triad in colorectal cancer microenvironment. Front Immunol. (2021) 12:612826. doi: 10.3389/fimmu.2021.612826, PMID: 33841394 PMC8033001

[151] Makki K Deehan EC Walter J Bäckhed F. The impact of dietary fiber on gut microbiota in host health and disease. Cell Host Microbe. (2018) 23:705–15. doi: 10.1016/j.chom.2018.05.012, PMID: 29902436

[152] Yang W Yu T Huang X Bilotta AJ Xu L Lu Y. Intestinal microbiota-derived short-chain fatty acids regulation of immune cell IL-22 production and gut immunity. Nat Commun. (2020) 11:4457. doi: 10.1038/s41467-020-18262-6, PMID: 32901017 PMC7478978

[153] Zhu X Li K Liu G Wu R Zhang Y Wang S. Microbial metabolite butyrate promotes anti-PD-1 antitumor efficacy by modulating T cell receptor signaling of cytotoxic CD8 T cell. Gut Microbes. (2023) 15:2249143. doi: 10.1080/19490976.2023.2249143, PMID: 37635362 PMC10464552

[154] Bachem A Makhlouf C Binger KJ de Souza DP Tull D Hochheiser K. Microbiota-derived short-chain fatty acids promote the memory potential of antigen-activated CD8(+) T cells. Immunity. (2019) 51:285–97.e5. doi: 10.1016/j.immuni.2019.06.002, PMID: 31272808

[155] Donohoe DR Holley D Collins LB Montgomery SA Whitmore AC Hillhouse A. A gnotobiotic mouse model demonstrates that dietary fiber protects against colorectal tumorigenesis in a microbiota- and butyrate-dependent manner. Cancer Discov. (2014) 4:1387–97. doi: 10.1158/2159-8290.CD-14-0501, PMID: 25266735 PMC4258155

[156] Li Q Cao L Tian Y Zhang P Ding C Lu W. Butyrate suppresses the proliferation of colorectal cancer cells via targeting pyruvate kinase M2 and metabolic reprogramming. Mol Cell Proteomics. (2018) 17:1531–45. doi: 10.1074/mcp.RA118.000752, PMID: 29739823 PMC6072541

[157] Bekebrede AF Deuren TV Gerrits WJJ Keijer J Boer VCJ. Butyrate alters pyruvate flux and induces lipid accumulation in cultured colonocytes. Int J Mol Sci. (2021) 22:10937. doi: 10.3390/ijms222010937, PMID: 34681598 PMC8539916

[158] Huang C Deng W Xu HZ Zhou C Zhang F Chen J. Short-chain fatty acids reprogram metabolic profiles with the induction of reactive oxygen species production in human colorectal adenocarcinoma cells. Comput Struct Biotechnol J. (2023) 21:1606–20. doi: 10.1016/j.csbj.2023.02.022, PMID: 36874158 PMC9975252

[159] Schug ZT Vande Voorde J Gottlieb E. The metabolic fate of acetate in cancer. Nat Rev Cancer. (2016) 16:708–17. doi: 10.1038/nrc.2016.87, PMID: 27562461 PMC8992383

[160] Qiu J Villa M Sanin DE Buck MD O'Sullivan D Ching R. Acetate promotes T cell effector function during glucose restriction. Cell Rep. (2019) 27:2063–74.e5. doi: 10.1016/j.celrep.2019.04.022, PMID: 31091446 PMC6544383

[161] Depner M Taft DH Kirjavainen PV Kalanetra KM Karvonen AM Peschel S. Maturation of the gut microbiome during the first year of life contributes to the protective farm effect on childhood asthma. Nat Med. (2020) 26:1766–75. doi: 10.1038/s41591-020-1095-x, PMID: 33139948

[162] Ryu TY Kim K Han TS Lee MO Lee J Choi J. Human gut-microbiome-derived propionate coordinates proteasomal degradation via HECTD2 upregulation to target EHMT2 in colorectal cancer. Isme J. (2022) 16:1205–21. doi: 10.1038/s41396-021-01119-1, PMID: 34972816 PMC9038766

[163] Nshanian M Gruber JJ Geller BS Chleilat F Lancaster SM White SM. Short-chain fatty acid metabolites propionate and butyrate are unique epigenetic regulatory elements linking diet, metabolism and gene expression. Nat Metab. (2025) 7:196–211. doi: 10.1038/s42255-024-01191-9, PMID: 39789354 PMC11774759

[164] Sinha SR Haileselassie Y Nguyen LP Tropini C Wang M Becker LS. Dysbiosis-induced secondary bile acid deficiency promotes intestinal inflammation. Cell Host Microbe. (2020) 27:659–70.e5. doi: 10.1016/j.chom.2020.01.021, PMID: 32101703 PMC8172352

[165] Ocvirk S O'Keefe SJD. Dietary fat, bile acid metabolism and colorectal cancer. Semin Cancer Biol. (2021) 73:347–55. doi: 10.1016/j.semcancer.2020.10.003, PMID: 33069873

[166] Zhang H Xu H Zhang C Tang Q Bi F. Ursodeoxycholic acid suppresses the Malignant progression of colorectal cancer through TGR5-YAP axis. Cell Death Discov. (2021) 7:207. doi: 10.1038/s41420-021-00589-8, PMID: 34365464 PMC8349355

[167] Yang J Wei H Zhou Y Szeto CH Li C Lin Y. High-fat diet promotes colorectal tumorigenesis through modulating gut microbiota and metabolites. Gastroenterology. (2022) 162:135–49.e2. doi: 10.1053/j.gastro.2021.08.041, PMID: 34461052

[168] Li J Chen Z Wang Q Du L Yang Y Guo F. Microbial and metabolic profiles unveil mutualistic microbe-microbe interaction in obesity-related colorectal cancer. Cell Rep Med. (2024) 5:101429. doi: 10.1016/j.xcrm.2024.101429, PMID: 38378003 PMC10982962

[169] Jun SY Brown AJ Chua NK Yoon JY Lee JJ Yang JO. Reduction of squalene epoxidase by cholesterol accumulation accelerates colorectal cancer progression and metastasis. Gastroenterology. (2021) 160:1194–207.e28. doi: 10.1053/j.gastro.2020.09.009, PMID: 32946903

[170] Li C Wang Y Liu D Wong CC Coker OO Zhang X. Squalene epoxidase drives cancer cell proliferation and promotes gut dysbiosis to accelerate colorectal carcinogenesis. Gut. (2022) 71:2253–65. doi: 10.1136/gutjnl-2021-325851, PMID: 35232776 PMC9554078

[171] Pan M Qin C Han X. Lipid metabolism and lipidomics applications in cancer research. Adv Exp Med Biol. (2021) 1316:1–24. doi: 10.1007/978-981-33-6785-2_1., PMID: 33740240 PMC8287890

[172] Juloski T Popovic T Martacic JD Cuk V Milic Perovic Stankovic S. Fatty acid in colorectal cancer in adult and aged patients of both sexes. J buon. (2021) 26:1898–907. J TJV VC, M SMP, M SS., PMID: 34761598

[173] Bhatt K Orlando T Meuwis MA Louis E Stefanuto PH Focant JF. Comprehensive insight into colorectal cancer metabolites and lipids for human serum: A proof-of-concept study. Int J Mol Sci. (2023) 24:9614. doi: 10.3390/ijms24119614, PMID: 37298566 PMC10253775

[174] Mika A Pakiet A Czumaj A Kaczynski Z Liakh I Kobiela J. Decreased triacylglycerol content and elevated contents of cell membrane lipids in colorectal cancer tissue: A lipidomic study. J Clin Med. (2020) 9:1095. doi: 10.3390/jcm9041095, PMID: 32290558 PMC7230725

[175] Tan B Qiu Y Zou X Chen T Xie G Cheng Y. Metabonomics identifies serum metabolite markers of colorectal cancer. J Proteome Res. (2013) 12:3000–9. doi: 10.1021/pr400337b, PMID: 23675754 PMC5902797

[176] Dmitrieva-Posocco O Wong AC Lundgren P Golos AM Descamps HC Dohnalová L. β-Hydroxybutyrate suppresses colorectal cancer. Nature. (2022) 605:160–5. doi: 10.1038/s41586-022-04649-6, PMID: 35477756 PMC9448510

[177] Liu T Peng F Yu J Tan Z Rao T Chen Y. LC-MS-based lipid profile in colorectal cancer patients: TAGs are the main disturbed lipid markers of colorectal cancer progression. Anal Bioanal Chem. (2019) 411:5079–88. doi: 10.1007/s00216-019-01872-5, PMID: 31201454

[178] Fang Z He M Song M. Serum lipid profiles and risk of colorectal cancer: a prospective cohort study in the UK Biobank. Br J Cancer. (2021) 124:663–70. doi: 10.1038/s41416-020-01143-6, PMID: 33139801 PMC7851156

[179] Elmallah MIY Ortega-Deballon P Hermite L Pais-De-Barros JP Gobbo J Garrido C. Lipidomic profiling of exosomes from colorectal cancer cells and patients reveals potential biomarkers. Mol Oncol. (2022) 16:2710–8. doi: 10.1002/1878-0261.13223, PMID: 35524452 PMC9298677

[180] Zhu Y Zhou H Chen H Zhang J Liang Y Yang S. Global serum metabolomic and lipidomic analyses reveal lipid perturbations and potential biomarkers of the colorectal cancer by adenoma-carcinoma sequence. Chin J Analytical Chem. (2023) 51:100270. doi: 10.1016/j.cjac.2023.100270

[181] Xu R Shen J Song Y Lu J Liu Y Cao Y. Exploration of the application potential of serum multi-biomarker model in colorectal cancer screening. Sci Rep. (2024) 14:10127. doi: 10.1038/s41598-024-60867-0, PMID: 38698075 PMC11066011

[182] Cajka T Fiehn O. Comprehensive analysis of lipids in biological systems by liquid chromatography-mass spectrometry. Trends Analyt Chem. (2014) 61:192–206. doi: 10.1016/j.trac.2014.04.017, PMID: 25309011 PMC4187118

[183] Lubes G Goodarzi M. GC-MS based metabolomics used for the identification of cancer volatile organic compounds as biomarkers. J Pharm BioMed Anal. (2018) 147:313–22. doi: 10.1016/j.jpba.2017.07.013, PMID: 28750734

[184] Hu Z Shen F Liu Y Zhong Z Chen Y Xia Z. Targeted metabolomics reveals novel diagnostic biomarkers for colorectal cancer. Mol Oncol. (2025) 19:1737–50. doi: 10.1002/1878-0261.13791, PMID: 39753208 PMC12161463

[185] Maimó-Barceló A Pérez-Romero K Rodríguez RM Huergo C Calvo I Fernández JA. To image or not to image: Use of imaging mass spectrometry in biomedical lipidomics. Prog Lipid Res. (2025) 97:101319. doi: 10.1016/j.plipres.2025.101319, PMID: 39765282

[186] Denti V Mahajneh A Capitoli G Clerici F Piga I Pagani L. Lipidomic typing of colorectal cancer tissue containing tumour-infiltrating lymphocytes by MALDI mass spectrometry imaging. Metabolites. (2021) 11:599. doi: 10.3390/metabo11090599, PMID: 34564418 PMC8471593

[187] Dufresne M Fincher JA Patterson NH Schey KL Norris JL Caprioli RM. α-cyano-4-hydroxycinnamic acid and tri-potassium citrate salt pre-coated silicon nanopost array provides enhanced lipid detection for high spatial resolution MALDI imaging mass spectrometry. Anal Chem. (2021) 93:12243–9. doi: 10.1021/acs.analchem.1c01560, PMID: 34449196

[188] Iakab SA Ràfols P Correig-Blanchar X García-Altares M. Perspective on multimodal imaging techniques coupling mass spectrometry and vibrational spectroscopy: picturing the best of both worlds. Anal Chem. (2021) 93:6301–10. doi: 10.1021/acs.analchem.0c04986, PMID: 33856207 PMC8491157

[189] Yin R Burnum-Johnson KE Sun X Dey SK Laskin J. High spatial resolution imaging of biological tissues using nanospray desorption electrospray ionization mass spectrometry. Nat Protoc. (2019) 14:3445–70. doi: 10.1038/s41596-019-0237-4, PMID: 31723300 PMC6941488

[190] Passarelli MK Pirkl A Moellers R Grinfeld D Kollmer F Havelund R. The 3D OrbiSIMS—label-free metabolic imaging with subcellular lateral resolution and high mass-resolving power. Nat Methods. (2017) 14:1175–83. doi: 10.1038/nmeth.4504, PMID: 29131162

[191] Li J Vosegaard T Guo Z. Applications of nuclear magnetic resonance in lipid analyses: An emerging powerful tool for lipidomics studies. Prog Lipid Res. (2017) 68:37–56. doi: 10.1016/j.plipres.2017.09.003, PMID: 28911967

[192] Batchuluun B Pinkosky SL Steinberg GR. Lipogenesis inhibitors: therapeutic opportunities and challenges. Nat Rev Drug Discovery. (2022) 21:283–305. doi: 10.1038/s41573-021-00367-2, PMID: 35031766 PMC8758994

[193] Chang L Wu P Senthilkumar R Tian X Liu H Shen X. Loss of fatty acid synthase suppresses the Malignant phenotype of colorectal cancer cells by down-regulating energy metabolism and mTOR signaling pathway. J Cancer Res Clin Oncol. (2016) 142:59–72. doi: 10.1007/s00432-015-2000-8, PMID: 26109148 PMC11819371

[194] Murata S Yanagisawa K Fukunaga K Oda T Kobayashi A Sasaki R. Fatty acid synthase inhibitor cerulenin suppresses liver metastasis of colon cancer in mice. Cancer Sci. (2010) 101:1861–5. doi: 10.1111/j.1349-7006.2010.01596.x, PMID: 20491775 PMC11159773

[195] Khiewkamrop P Surangkul D Srikummool M Richert L Pekthong D Parhira S. Epigallocatechin gallate triggers apoptosis by suppressing *de novo* lipogenesis in colorectal carcinoma cells. FEBS Open Bio. (2022) 12:937–58. doi: 10.1002/2211-5463.13391, PMID: 35243817 PMC9063442

[196] Shin CM Lee DH Seo AY Lee HJ Kim SB Son WC. Green tea extracts for the prevention of metachronous colorectal polyps among patients who underwent endoscopic removal of colorectal adenomas: A randomized clinical trial. Clin Nutr. (2018) 37:452–8. doi: 10.1016/j.clnu.2017.01.014, PMID: 28209333

[197] Nalli M Masci D Urbani A La Regina G Silvestri R. Emerging direct targeting β-catenin agents. Molecules. (2022) 27:7735. doi: 10.3390/molecules27227735, PMID: 36431838 PMC9698307

[198] Yao Y Rao C Zheng G Wang S. Luteolin suppresses colorectal cancer cell metastasis via regulation of the miR−384/pleiotrophin axis. Oncol Rep. (2019) 42:131–41. doi: 10.3892/or.2019.7136, PMID: 31059061 PMC6549074

[199] Falchook G Infante J Arkenau HT Patel MR Dean E Borazanci E. First-in-human study of the safety, pharmacokinetics, and pharmacodynamics of first-in-class fatty acid synthase inhibitor TVB-2640 alone and with a taxane in advanced tumors. EClinicalMedicine. (2021) 34:100797. doi: 10.1016/j.eclinm.2021.100797, PMID: 33870151 PMC8040281

[200] Cioccoloni G Bonmassar L Pagani E Caporali S Fuggetta MP Bonmassar E. Influence of fatty acid synthase inhibitor orlistat on the DNA repair enzyme O6-methylguanine-DNA methyltransferase in human normal or Malignant cells *in vitro* . Int J Oncol. (2015) 47:764–72. doi: 10.3892/ijo.2015.3025, PMID: 26035182

[201] Hatzivassiliou G Zhao F Bauer DE Andreadis C Shaw AN Dhanak D. ATP citrate lyase inhibition can suppress tumor cell growth. Cancer Cell. (2005) 8:311–21. doi: 10.1016/j.ccr.2005.09.008, PMID: 16226706

[202] Wang C Xu C Sun M Luo D Liao DF Cao D. Acetyl-CoA carboxylase-alpha inhibitor TOFA induces human cancer cell apoptosis. Biochem Biophys Res Commun. (2009) 385:302–6. doi: 10.1016/j.bbrc.2009.05.045, PMID: 19450551 PMC2724073

[203] Potze L di Franco S Kessler JH Stassi G Medema JP. Betulinic acid kills colon cancer stem cells. Curr Stem Cell Res Ther. (2016) 11:427–33. doi: 10.2174/1574888X11666151203223512, PMID: 26647913

[204] Hernlund E Ihrlund LS Khan O Ates YO Linder S Panaretakis T. Potentiation of chemotherapeutic drugs by energy metabolism inhibitors 2-deoxyglucose and etomoxir. Int J Cancer. (2008) 123:476–83. doi: 10.1002/ijc.v123:2, PMID: 18452174

[205] Shiragami R Murata S Kosugi C Tezuka T Yamazaki M Hirano A. Enhanced antitumor activity of cerulenin combined with oxaliplatin in human colon cancer cells. Int J Oncol. (2013) 43:431–8. doi: 10.3892/ijo.2013.1978, PMID: 23754252

[206] Toden S Tran HM Tovar-Camargo OA Okugawa Y Goel A. Epigallocatechin-3-gallate targets cancer stem-like cells and enhances 5-fluorouracil chemosensitivity in colorectal cancer. Oncotarget. (2016) 7:16158–71. doi: 10.18632/oncotarget.v7i13, PMID: 26930714 PMC4941304

[207] Zhou Y Bollu LR Tozzi F Ye X Bhattacharya R Gao G. ATP citrate lyase mediates resistance of colorectal cancer cells to SN38. Mol Cancer Ther. (2013) 12:2782–91. doi: 10.1158/1535-7163.MCT-13-0098, PMID: 24132143 PMC4302275

[208] Zi R Zhao X Liu L Wang Y Zhang R Bian Z. Metabolic-immune suppression mediated by the SIRT1-CX3CL1 axis induces functional enhancement of regulatory T cells in colorectal carcinoma. Adv Sci (Weinh). (2025):e2404734. doi: 10.1002/advs.202404734, PMID: 39783838 PMC12061293

[209] Masci D Puxeddu M Silvestri R La Regina G. Metabolic rewiring in cancer: small molecule inhibitors in colorectal cancer therapy. Molecules. (2024) 29:2110. doi: 10.3390/molecules29092110, PMID: 38731601 PMC11085455

[210] Schiliro C Firestein BL. Mechanisms of metabolic reprogramming in cancer cells supporting enhanced growth and proliferation. Cells. (2021) 10:3593. doi: 10.3390/cells10051056, PMID: 33946927 PMC8146072

